# A Finite Element Model for Mixed Porohyperelasticity with Transport, Swelling, and Growth

**DOI:** 10.1371/journal.pone.0152806

**Published:** 2016-04-14

**Authors:** Michelle Hine Armstrong, Adrián Buganza Tepole, Ellen Kuhl, Bruce R. Simon, Jonathan P. Vande Geest

**Affiliations:** 1 Graduate Interdisciplinary Program in Applied Mathematics, The University of Arizona, Tucson, AZ, United States of America; 2 Department of Mechanical Engineering, Stanford University, Stanford, CA, United States of America; 3 Department of Aerospace and Mechanical Engineering, The University of Arizona, Tucson, AZ, United States of America; 4 Graduate Interdisciplinary Program of Biomedical Engineering, The University of Arizona, Tucson, AZ, United States of America; 5 BIO5 Institute for Biocollaborative Research, The University of Arizona, Tucson, AZ 85721, United States of America; 6 Department of Biomedical Engineering, The University of Arizona, Tucson, AZ 85721, United States of America; 7 Department of Bioengineering, The University of Pittsburgh, Pittsburgh, PA 15219, United States of America; University of Washington, UNITED STATES

## Abstract

The purpose of this manuscript is to establish a unified theory of porohyperelasticity with transport and growth and to demonstrate the capability of this theory using a finite element model developed in MATLAB. We combine the theories of volumetric growth and mixed porohyperelasticity with transport and swelling (MPHETS) to derive a new method that models growth of biological soft tissues. The conservation equations and constitutive equations are developed for both solid-only growth and solid/fluid growth. An axisymmetric finite element framework is introduced for the new theory of growing MPHETS (GMPHETS). To illustrate the capabilities of this model, several example finite element test problems are considered using model geometry and material parameters based on experimental data from a porcine coronary artery. Multiple growth laws are considered, including time-driven, concentration-driven, and stress-driven growth. Time-driven growth is compared against an exact analytical solution to validate the model. For concentration-dependent growth, changing the diffusivity (representing a change in drug) fundamentally changes growth behavior. We further demonstrate that for stress-dependent, solid-only growth of an artery, growth of an MPHETS model results in a more uniform hoop stress than growth in a hyperelastic model for the same amount of growth time using the same growth law. This may have implications in the context of developing residual stresses in soft tissues under intraluminal pressure. To our knowledge, this manuscript provides the first full description of an MPHETS model with growth. The developed computational framework can be used in concert with novel in-vitro and in-vivo experimental approaches to identify the governing growth laws for various soft tissues.

## Introduction

The theories of mixed porohyperelasticity and growth have developed separately, and our laboratory has been working to unite them into one combined theory. Porohyperelasticity and biphasic/triphasic theory have long been used to describe the biomechanical response of hard and soft tissues [1–4]. Meanwhile, the theory of growth and remodeling has been used to describe things as varied as the evolution of bone and shells [5], heart [6], airways [7], arteries [8], skin [9], muscle [10], tumors [11], and eyes [12]. In this paper we will combine the theory of mixed porohyperelasticity with transport and swelling (MPHETS) [1, 3, 13–15] with volumetric growth [6, 16–18].

### Modeling multiphasic soft tissues

Many soft tissues consist of a porous solid skeleton that is fully saturated by an interstitial fluid and as such can be adequately modled as a fully saturated porohyperelastic (PHE) material. Furthermore, one may also wish to track the behavior of a dissolved species representing a drug, growth factor, or naturally occurring cytokines, which can be done utilizing a few different approaches [1–4, 19–23].

The theory of saturated porous media was first developed to describe soil mechanics [24–26] and has served as the background for applications to many problems in biomechanics. This background resulted in the creation of multiple theoretical models to describe soft tissue as a fully saturated porous media [2, 13, 20, 27]. Other similar approaches were also developed to model soft tissues as triphasic materials [3, 4, 21, 22] and multiphasic materials with a charged chemical species [1, 19, 23, 28–30].

### Volumetric growth

The theory of growth and remodeling is rooted in the theory of elastoplasticity [31], which was further applied to describe biological growth [5, 16, 32–34]. Two main theories emerged to describe growth and remodeling. The first, which we follow in this paper, is volumetric growth [6, 16–18, 35]. The second theoretical framework to describe growth and remodeling is via a constrained mixture model [36–38].

#### Growth versus remodeling

In practice, some growth law is assigned to the system which causes material addition or resorption. In the case of growth, the newly added material may or may not have the same properties (e.g., density, elastic modulus) as the old material. Note that there is some disagreement about what actually constitutes remodeling. The term “remodeling” may be used to describe situations where the material properties change; e.g., the new material is more dense than the original material. “Remodeling” also may be used to describe situations where the material parameters are the same, but the system behavior changes. For example, the addition of material to an artery causes the artery to be thicker, and hence, stiffer. However, the material properties themselves remain the same.

For this work, we adopt the definitions that “growth” (or resorption) will indicate matter with the same material properties being added (or resorbed) while “remodeling” will indicate a change in density or other properties of the material. The combination of the two, “growth and remodeling,” will indicate a change in the volume as well as a change in material properties [17]. For the current work, we assume constant density, and therefore only consider the case of growth.

### Growth and MPHETS

Our laboratory recently developed a preliminary one-dimensional model incorporating both the theories of porohyperelasticity with transport and swelling as well as stress-dependent growth [39, 40]. That first approach used a simplistic coupling of stress-dependent growth of the solid based on work from [6, 17]. For illustration of the theory, Harper et al.’s approach was formulated in one dimension [40].

The present work expands on the model presented in Harper et al. [40], and incorporates growth based on time, chemical concentration, and stress, rather than just stress. The current theory is more sophisticated than the model presented in [40]. In this manuscript, we explicitly calculate the grown masses of the solid and the fluid as well as the porosity change due to growth, which allows us to include the effect of growth on bulk fluid motion and chemical concentration by introducing volumetric source terms. The equations in the tangent modulus are more fully developed, and include several linear and nonlinear growth models depending on time and concentration in addition to mechanical stress. Furthermore, the current theory is developed for a general coordinate system and implemented in axisymmetry, and so is better equipped to handle more anatomically complicated biological problems.

To our knowledge, there does not currently exist a growth and remodeling framework that brings together the theories of volumetric growth and mixed porohyperelasticity with transport and swelling. Therefore, the purpose of this manuscript is to develop a theoretical foundation for growth of soft tissues utilizing a mixed porous media formulation, such that growth can be a function of time, mechanical stress, or chemical species concentration. In part, the novelty of this approach allows growth constitutive relations to be a function of large neutral mobile species, such that future growth models can be influenced by naturally occurring cytokines, growth factors, and/or pharmaceuticals in addition to mechanical stimuli. An axisymmetric finite element framework is introduced and several test problems are considered in the context of a coronary artery, including comparing the new growing porohyperelastic model with a traditional solid-only growth formulation.

## Materials and Methods

The current work is deeply rooted in the theories of MPHETS and volumetric growth. For space considerations, we have provided a brief summary of MPHETS theory in S1 Appendix. For more detail on MPHETS, the reader is referred to [1, 3, 13–15, 40, 41]. For further background on growth, we refer the reader to [5, 6, 16, 17, 42].

### Model assumptions

An MPHETS material is a continuum made up of a fully-saturated, incompressible porous solid; an incompressible interstitial fluid; and a neutral, mobile chemical species [15]. The primary variables are the displacement potential **u**, fluid potential ${\tilde{\mu }}^{f^{*}}$ and chemical potential ${\tilde{\mu }}^{c^{*}}$, where the tilde denotes that these are Lagrangian quantities. The secondary variables are the pore fluid pressure *p*^*f*^ and the species concentration *c*. Together, the constituents form a porohyperelastic compressible material: as the material is pressurized, the interstitial fluid flows, and the chemical species is diffused and convected within the deforming porous media. The chemical species may also induce flow via increases in osmotic pressure. Simulating the swelling and transport of a chemical species may help better understand cytokine, growth factor, and drug distribution in soft tissues [43].

Because our MPHETS model is meant to simulate in-vivo biomechanical behavior, we assume a constant temperature of 310 K. We also assume that all MPHETS transport parameters (porosity, convection coupling coefficient, and diffusivity) are isotropic in the Eulerian frame; they are related to the Lagrangian MPHETS parameters via the deformation (Eqs (65)-(67)). For illustration purposes, the transport parameters are further assumed to be constant in the example problems.

### Considerations for adding growth to an MPHETS model

The two cases of growth considered in this paper are growth of the solid constituent only, and growth of the porous material (addition of solid and fluid constituents). We assume density preservation; the density in the intermediate growth configuration will be the same as the initial density of the material. This density preservation will be discussed further below.

A fundamental constitutive equation for an MPHETS model is the effective stress principle, which breaks the second Piola-Kirchhoff stress into a fluid stress and an effective stress. The effective stress is defined as the stress that “has its seat exclusively in the solid phase of the [material]”; that is, the effective stress is the excess stress in the material after subtracting out the stress on the fluid constituent [44]. Then assuming an orthogonal curvilinear coordinate system, the second Piola-Kirchhoff stress tensor *S*_*ij*_ may be written in the Lagrangian frame as

$$\begin{array}{c} S_{ij}=S_{ij}^{\text{eff}}-JH_{ij}p^{f},\;S=S^{\text{eff}}-JHp^{f} \end{array}$$ (1)

where $S_{ij}^{\text{eff}}$ is the effective second Piola-Kirchhoff stress tensor, *J* is the Jacobian of the deformation gradient *F*_*ij*_, Finger’s strain tensor is defined as $H_{ij}=F_{ip}^{-1}F_{jp}^{-1}$, and *p*^*f*^ is the pore fluid pressure. We assume that stress-driven growth will depend on the effective mechanical stress.

In this manuscript, we consider three types of growth motivation: dependence on time (constant growth), dependence on concentration, and dependence on stress in the material. Growth dependent on time or concentration is a function of a scalar catalyst which has no directionality. For stress-dependent growth of a purely solid material, the trace of the Mandel stress is energy conjugate to the growth velocity gradient [17]; thus, the trace of the Mandel stress is an appropriate driver for growth. For stress-dependent growth of a porohyperelastic material, we choose to have the growth depend on the effective stress. The effective, elastic Mandel stress **M**^eff,*e*^, is defined as

$$\begin{array}{c} M_{ij}^{\text{eff},e}=C_{ik}^{e}S_{kj}^{\text{eff},e},\;M^{\text{eff},e}=C^{e}S^{\text{eff},e} \end{array}$$ (2)

where $C_{ij}^{e}=F_{pi}^{e}F_{pj}^{e}$ for elastic deformation gradient $F_{pi}^{e}$, defined below; and where $S_{kj}^{\text{eff},e}$ is the effective, elastic second Piola-Kirchhoff stress tensor, defined below. Note that the Mandel stress, as the product of two symmetric tensors, is also symmetric. Because the trace of the Mandel stress is a scalar, there is no directionality to this value. Thus, in all cases considered here, isotropic growth is appropriate.

### General theory for isotropic growth

As a precursor to adding growth to the MPHETS theory, in this section we summarize some general principles of isotropic growth including densities in the different configurations associated with growth. More detail is available in [6, 17].

**Fig 1:**
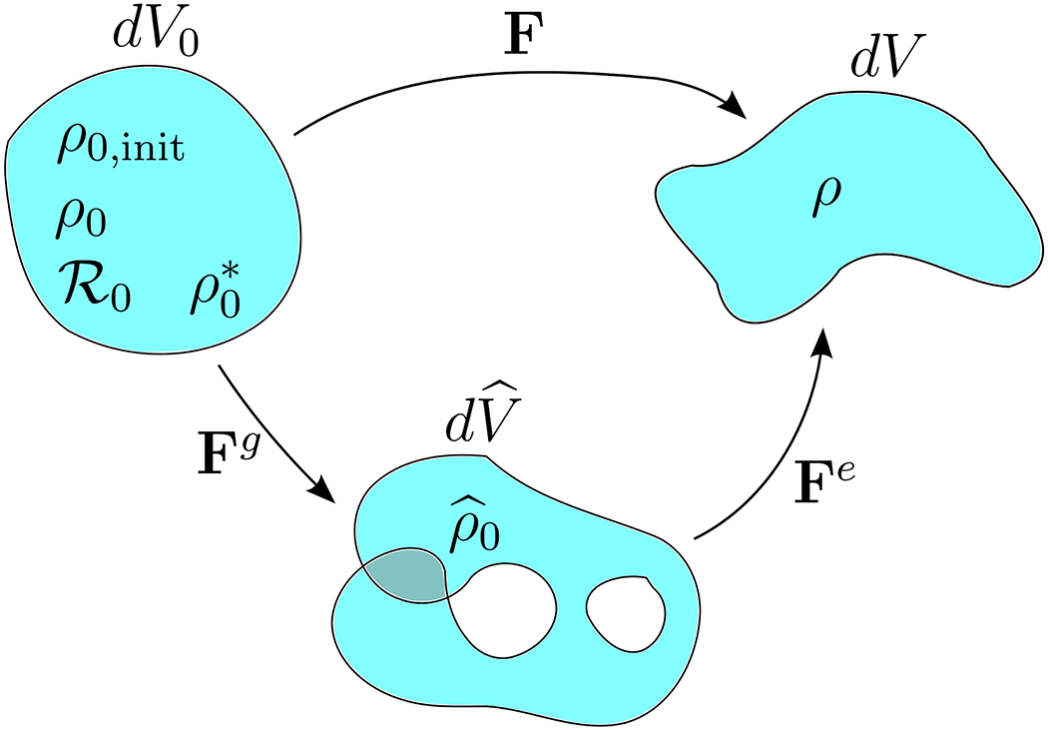
The total deformation gradient F maps the original material to the final grown, deformed state. The total deformation may be split via the multiplicative decomposition into a growth deformation **F**^*g*^ followed by an elastic deformation **F**^*e*^. The intermediate configuration may have holes or overlapping material. The original material has differential volume *dV*_0_ and initial density *ρ*_0,init_. The mass source $R_{0}$ causes a grown mass *dm* such that the new grown density in the reference configuration is given by *ρ*_0_ = *dm*/*dV*_0_. For the assumption of density preservation, $\rho_{0}^{*}$ is the density of the material that is preserved from the initial to the grown configuration. For a single (solid) constituent, $\rho_{0}^{*}=\rho_{0,\text{init}}.$ The intermediate growth configuration has differential volume $d\hat{V}$ and grown density ${\hat{\rho }}_{0}=dm/d\hat{V}$. The final (grown, deformed) configuration has differential volume *dV* and grown density *ρ* = *dm*/*dV*.

Total deformation may be split through the multiplicative decomposition into an elastic part **F**^*e*^ and a growth part **F**^*g*^ such that

$$\begin{array}{c} F=F^{e}F^{g}, \end{array}$$ (3)

as seen in Fig 1. The Jacobian of **F** is the product of the elastic and growth Jacobians, *J* = *J*^*e*^ *J*^*g*^.

By assumption, three configurations exist: (1) original reference configuration—a physical, possibly stressed configuration, (2) intermediate growth state—a non-physical, locally stress-free state, and (3) final configuration—a physical, both grown and loaded state, which may contain residual stresses.

For isotropic growth, the growth deformation gradient may be defined by **F**^*g*^ = *ϑ* **I**, where *ϑ* is a volumetric stretch. Then for isotropic growth, the elastic deformation may be written simply as **F**^*e*^ = **F**(**F**^*g*^)^−1^ = **F**/*ϑ*.

The growth law will be formulated as a rate that depends on some system variables; for example,

$$\begin{array}{c} \dot{\vartheta }=k_{\vartheta }\left(\vartheta \right)\phi^{g}\left(t,c,F,M^{\text{eff}},\ldots \right), \end{array}$$ (4)

where *k*_*ϑ*_ is a scalar, generally non-constant parameter and *ϕ*^*g*^ is some scalar-valued growth rate function of time *t*, concentration *c*, strain via the deformation gradient **F**, effective Mandel stress **M**^eff^, or other relevant variables.

There are five different densities associated with growth, as pictured in Fig 1. The different densities are used to calculate the growth source term. The initial material density is given by *ρ*_0,init_; this is defined as the initial mass *dM* divided by the original volume *dV*_0_:

$$\begin{array}{c} \rho_{0,\text{init}}=dM/dV_{0}. \end{array}$$ (5)

The grown density in the original configuration is represented by *ρ*_0_ and is given by the grown mass *dm* divided by the original volume. The grown density in the intermediate configuration and final configurations are denoted ${\hat{\rho }}_{0}$ and *ρ*, respectively. The grown densities in each configuration are related to each other via the Jacobians [17], such that

$$\begin{array}{c} \rho_{0}=dm/dV_{0}\overset{\text{d}}{=}J^{g}{\hat{\rho }}_{0}\overset{\text{d}}{=}J\rho . \end{array}$$ (6)

Note that in general, *ρ*_0_ ≠ *ρ*_0,init_ because the grown mass differs from the initial mass.

By the assumption of density preservation from the initial configuration to the intermediate configuration, the grown material is added at constant density denoted by $\rho_{0}^{*}$. All densities marked with a star will be held constant during growth. For traditional volumetric growth of a solid, there is only one initial density, so $\rho_{0}^{*}=\rho_{0,\text{init}}$. Then with the assumption of density preservation, the mass source $R_{0}$ may either be calculated as a function of the density change of the grown material in the reference configuration ${\dot{\rho }}_{0}$ or referred back to the initial density [17]. Then the mass source may be defined as

$$\begin{array}{c} R_{0}={\dot{\rho }}_{0}=3\rho_{0}^{*}\vartheta^{2}\dot{\vartheta }=3\rho_{0,\text{init}}\vartheta^{2}\dot{\vartheta }, \end{array}$$ (7)

as in [17]. Thus, given the time evolution of the scalar growth *ϑ*, we have completely determined the mass source from Eq (7) as a function of the initial density of the solid in the reference configuration and the scalar growth multiple *ϑ*.

Integrating the mass source term $R_{0}$ from *t*_0_ to *t* and multiplying by the reference volume will yield the differential mass growth element; we will use this technique below to determine the mass source for a porohyperelastic material.

### Source terms and final porosity for different growth cases of porohyperelasticity

For porohyperelasticity, the two non-negligible mass additions are the mass of the solid and the mass of the fluid. By assumption, the mass of the species may be neglected on the scale of the fluid and solid mass. We consider two types of growth: adding only solid material and adding a combination of solid and fluid. To determine the source terms, we must therefore carefully calculate the mass of the solid and the fluid during growth and elastic deformation. The following derivations follow the pattern laid out in Himpel et al. [17], but are calculated here for a porohyperelastic material.

#### Porosity and densities for MPHETS

An MPHETS material has multiple constituents and thus requires the definition of additional volumes and densities. The material is a combination of solid, fluid, and chemical species, but by assumption, the volume of the species can be neglected compared to the volumes of the solid and the fluid. Then the differential volume element for the material may be written as *dV* = *dV*^*s*^ + *dV*^*f*^ where *dV*^*s*^ is the differential volume of the solid and *dV*^*f*^ is the differential volume of the fluid. The porosity of the material gives the fraction of fluid volume to total volume in a specific frame. The Lagrangian porosity is defined as $n_{0}=dV_{0}^{f}/dV_{0}$ and the Eulerian porosity is defined as *n* = *dV*^*f*^/*dV*. The porosity may change because of total deformation and growth. An explicit relationship between the two porosity values will be derived below and presented in Eq (44).

A table of the different densities used in GMPHETS may be found in S6 Appendix. The true densities of the solid and the fluid are intrinsic properties and are defined as $\rho_{T}^{s}=dM^{s}/dV_{0}^{s}$ and $\rho_{T}^{f}=dM^{f}/dV_{0}^{f}$ for *dM*^*s*^ the initial mass of the solid and *dM*^*f*^ the initial mass of the fluid. For an incompressible constituent, the true density never changes and thus remains constant during the entire simulation. The true density of the fluid may be taken to be the density of water. The true density of the solid may be calculated in a two-step process: comparing the wet vs. dry weight from a purely collagenous material and using the dry volume of the collagen obtained through imaging.

In contrast, apparent densities change with the porosity. The apparent density of a constituent is defined as the mass of that constituent divided by the total volume, and may also be defined via the porosity *n*. For the initial apparent densities of the solid and the fluid, the definitions are given by $\rho_{0,\text{init}}^{s}=dM^{s}/dV_{0}=\left(1-n_{0}\right)\rho_{T}^{s}$ and $\rho_{0,\text{init}}^{f}=dM^{f}/dV_{0}=n_{0}\rho_{T}^{f}$. The solid and fluid densities that will be preserved during growth will be denoted as $\rho_{0}^{s^{*}}$ and $\rho_{0}^{f^{*}}$, where the star denotes density preservation. By assumption, adding solid-only material will preserve the true density of the solid ($\rho_{0}^{s^{*}}=\rho_{T}^{s}$) while adding a combination of solid and fluid will preserve the initial apparent densities of the solid and the fluid ($\rho_{0}^{s^{*}}=\rho_{0,\text{init}}^{s}$ and $\rho_{0}^{f^{*}}=\rho_{0,\text{init}}^{f}$).

#### The growth mass element

The grown mass of the solid *dm*^*s*^ can be calculated as

$$\begin{array}{c} dm^{s}=dM^{s}+\int_{t_{0}}^{t}R_{0}^{s}d\bar{t}dV_{0}, \end{array}$$ (8)

where *dM*^*s*^ is the initial mass of the solid at time *t*_0_ and $R_{0}^{s}$ is the Lagrangian volume-specific mass source of the solid, integrated from time *t*_0_ to *t*.

The mass of the fluid can be calculated by tracking the change in mass over time:

$$\begin{array}{c} dm^{f}=dM^{f}+\int_{t_{0}}^{t}R_{0}^{f}d\bar{t}dV_{0}-\Phi^{f}, \end{array}$$ (9)

where *dM*^*f*^ is the initial mass of the fluid, $R_{0}^{f}$ is the fluid’s Lagrangian volume-specific mass source, and Φ^*f*^ is the total mechanical flux of fluid from time *t*_0_ to *t*. Note that by assumption, this mechanical fluid flux is not associated with growth; any growth associated with the fluid must come from the source term $R_{0}^{f}$.

As in the general case, the grown apparent densities for constituent *α* in each configuration are related to each other via the Jacobians, such that

$$\begin{array}{c} \rho_{0}^{\alpha }=J^{g}{\hat{\rho }}_{0}^{\alpha }=J\rho^{\alpha },\;\text{for}\;\alpha \in \{s,f\}, \end{array}$$ (10)

where $\rho_{0}^{\alpha }=dm^{\alpha }/dV_{0}$, ${\hat{\rho }}_{0}^{\alpha }=dm^{\alpha }/d\hat{V}$, and *ρ*^*α*^ = *dm*^*α*^/*dV*. A table of the different densities used in GMPHETS may be found in S6 Appendix. Recall that the volumetric deformation *J*^*g*^ does not include flux because by assumption the growth deformation **F**^*g*^ does not include flux; in this derivation we only consider growth through source terms and not through mass flux. In contrast, the total deformation *J* does include the effects of flux because the total deformation gradient **F** includes fluid flux via mechanical effects from the elastic deformation.

For simplicity of notation, we also define the masses added during growth for the solid and the fluid as

$$\begin{array}{c} dm^{s,g}=\int_{t_{0}}^{t}R_{0}^{s}d\bar{t}dV_{0},\;dm^{f,g}=\int_{t_{0}}^{t}R_{0}^{f}d\bar{t}dV_{0}. \end{array}$$ (11)

#### Solid-only growth

If we have growth of only the solid, there is only one source term $R_{0}^{s}$. We assume that during growth, the true density of the solid $\rho_{T}^{s}$ is preserved.

Dividing Eq (8) by the reference volume *dV*_0_, the grown apparent density of the solid in the original configuration $\rho_{0}^{s}$ can be written as

$$\begin{array}{c} \rho_{0}^{s}=\rho_{0,\text{init}}^{s}+\int_{t_{0}}^{t}R_{0}^{s}d\bar{t}, \end{array}$$ (12)

where $\rho_{0,\text{init}}^{s}$ is the initial apparent density of the solid at time *t*_0_.

Taking the time derivative, the growth rate of the solid density in the original configuration is given by

$$\begin{array}{c} {\dot{\rho }}_{0}^{s}=R_{0}^{s}. \end{array}$$ (13)

Following Himpel et al. [17], we use the relationships between the different densities (see Eq (10)) to refer the local balance of mass to the intermediate growth configuration. Then

$$\begin{array}{c} {\dot{\rho }}_{0}^{s}=\dot{\overset{︵}{J^{g}{\hat{\rho }}_{0}^{s}}}={\dot{J}}^{g}{\hat{\rho }}_{0}^{s}+J^{g}\dot{{\hat{\rho }}_{0}^{s}}=R_{0}^{s}. \end{array}$$ (14)

For an arbitrary value of ${\hat{\rho }}_{0}^{s}$, Eq (14) simplifies to yield

$$\begin{array}{c} R_{0}^{s}=\dot{J^{g}}{\hat{\rho }}_{0}^{s}+J^{g}\dot{{\hat{\rho }}_{0}^{s}}=J^{g}\text{tr}{\hat{L}}^{g}{\hat{\rho }}_{0}^{s}+J^{g}\dot{{\hat{\rho }}_{0}^{s}}, \end{array}$$ (15)

since $\dot{J^{g}}=J^{g}\text{tr}{\hat{L}}^{g}$ where ${\hat{L}}^{g}={\dot{F}}^{g}F^{g-1}$ [17]. For isotropic growth, we additionally have that ${\hat{L}}^{g}=\left(\dot{\vartheta }/\vartheta \right)I$ and *J*^*g*^ = *ϑ*^3^; then Eq (15) simplifies to

$$\begin{array}{c} R_{0}^{s}=\left(\vartheta^{3}\right)\left(3\dot{\vartheta }/\vartheta \right)\left({\hat{\rho }}_{0}^{s}\right)+\vartheta^{3}\dot{{\hat{\rho }}_{0}^{s}}=3\vartheta^{2}\dot{\vartheta }{\hat{\rho }}_{0}^{s}+\vartheta^{3}\dot{{\hat{\rho }}_{0}^{s}} \end{array}$$ (16)

With the assumption of solid-only growth, the porosity in the intermediate growth configuration will be different from the original configuration. Thus, we define the porosity in the intermediate growth configuration as $\hat{n}=d{\hat{V}}^{f}/d\hat{V},$ where $d{\hat{V}}^{f}$ is the differential volume of the fluid in the intermediate growth configuration. For solid-only growth, the fluid volume will be the same in the original and grown configurations ($dV_{0}^{f}=d{\hat{V}}^{f}$; recall that by assumption no mass flux occurs during growth). Then also using the definition of the growth Jacobian, the growth porosity for solid-only growth may be written simply in terms of the initial porosity and the growth multiplier:

$$\begin{array}{c} \hat{n}=d{\hat{V}}^{f}/d\hat{V}=dV_{0}^{f}/\left(J^{g}dV_{0}\right)=n_{0}/\vartheta^{3}. \end{array}$$ (17)

The time derivative of Eq (17) is given by $\dot{\hat{n}}=-3n_{0}\dot{\vartheta }/\vartheta^{4}.$

The growth porosity from Eq (17) may be used to define the intermediate grown apparent density of the solid, where by definition

$$\begin{array}{c} {\hat{\rho }}_{0}^{s}\overset{\text{d}}{=}\frac{dm^{s}}{d\hat{V}}=\frac{dm^{s}}{d{\hat{V}}^{s}}\frac{d{\hat{V}}^{s}}{d\hat{V}}=\frac{dm^{s}}{d{\hat{V}}^{s}}\left(1-\hat{n}\right). \end{array}$$ (18)

For solid-only growth, we assume that the true density of the solid is preserved in the growth configuration, so $dm^{s}/d{\hat{V}}^{s}=\rho_{T}^{s}$. Then the grown apparent density of the solid in the intermediate configuration may be written as

$$\begin{array}{c} {\hat{\rho }}_{0}^{s}=\rho_{T}^{s}\left(1-\hat{n}\right). \end{array}$$ (19)

Using this definition for the intermediate apparent solid density, Eq (16) becomes

$$\begin{array}{c} R_{0}^{s}=3\vartheta^{2}\dot{\vartheta }\rho_{T}^{s}\left(1-\hat{n}\right)+\vartheta^{3}\dot{\overset{︵}{\rho_{T}^{s}\left(1-\hat{n}\right)}}=3\vartheta^{2}\dot{\vartheta }\rho_{T}^{s}\left(1-\hat{n}\right)-\vartheta^{3}\rho_{T}^{s}\dot{\hat{n}}. \end{array}$$ (20)

Then plugging the growth porosity (Eq (17)) and its derivative into Eq (20),

$$\begin{array}{c} R_{0}^{s}=3\vartheta^{2}\dot{\vartheta }\rho_{T}^{s}(1-\frac{n_{0}}{\vartheta^{3}})-\vartheta^{3}\rho_{T}^{s}(\frac{-3n_{0}\dot{\vartheta }}{\vartheta^{4}})=3\vartheta^{2}\dot{\vartheta }\rho_{T}^{s}-3\vartheta^{-1}\dot{\vartheta }\rho_{T}^{s}n_{0}+3\vartheta^{-1}\rho_{T}^{s}\dot{\vartheta }n_{0}. \end{array}$$ (21)

Finally, simplifying Eq (21) yields the solid growth source term

$$\begin{array}{c} R_{0}^{s}=3\rho_{0}^{s^{*}}\vartheta^{2}\dot{\vartheta }=3\rho_{T}^{s}\vartheta^{2}\dot{\vartheta }. \end{array}$$ (22)

Note that this derivation is similar to the general theory for isotropic growth. However, while the true density of the solid is preserved by growth, the apparent density is not preserved. For continuity with the theory from Himpel et al. [17], we write the Lagrangian density that is preserved during growth as $\rho_{0}^{s^{*}}=\rho_{T}^{s}$.

Integrating the solid source from *t*_0_ to *t*, multiplying by the reference volume *dV*_0_, and assuming that *ϑ*(*t*_0_) = 1 (thus **F**^*g*^(*t*_0_) = **I**, so the initial condition is no growth) yields the added mass of the solid from growth

$$\begin{array}{c} dm^{s,g}=\int_{t_{0}}^{t}R_{0}^{s}d\bar{t}dV_{0}=\rho_{T}^{s}\underset{\text{solid}\;\text{volume}\;\text{added}\;\text{by}\;\text{growth}}{\underset{︸}{\left(\vartheta^{3}-1\right)dV_{0}}}. \end{array}$$ (23)

The density-specific solid source term will be used in conservation of solid mass, and is given by

$$\begin{array}{c} \frac{R_{0}^{s}}{\rho_{T}^{s}}=\frac{3\rho_{T}^{s}\vartheta^{2}\dot{\vartheta }}{\rho_{T}^{s}}=3\vartheta^{2}\dot{\vartheta }. \end{array}$$ (24)

#### Solid and fluid growth that preserves apparent densities

By assumption, solid and fluid growth will preserve the initial apparent densities of both solid and fluid constituents. Thus, solid growth will occur at constant growth density $\rho_{0}^{s^{*}}=\left(1-n_{0}\right)\rho_{T}^{s}$ while fluid growth occurs at constant growth density $\rho_{0}^{f^{*}}=n_{0}\rho_{T}^{f}$.

The grown material density in the original configuration is defined as the total grown mass divided by reference volume *dV*_0_. The total grown mass *dm*^*g*^ is the sum of the grown mass of the solid (*dM*^*s*^ + *dm*^*s*,*g*^) and the grown mass of the fluid (*dM*^*f*^ + *dm*^*f*,*g*^). Note that the grown mass of the fluid does not contain a flux term, because by assumption there is no mass flux during growth. (Fluid flux only occurs during elastic deformation.) Then the Lagrangian density of the whole material after growth may be written as

$$\begin{array}{c} \rho_{0}=\frac{dm^{g}}{dV_{0}}=\frac{\left(dM^{s}+dm^{s,g}\right)+\left(dM^{f}+dm^{f,g}\right)}{dV_{0}}. \end{array}$$ (25)

The grown material density can also be written as

$$\begin{array}{c} \rho_{0}=\rho_{0,\text{init}}+\int_{t_{0}}^{t}R_{0}d\bar{t}, \end{array}$$ (26)

where *ρ*_0,init_ is the initial (pre-growth) density in the undeformed configuration and $R_{0}$ is a Lagrangian source term.

The total material density after growth may be split in terms of the fluid and solid (without flux, because by assumption no flux occurs during the growth process). The grown apparent densities of the solid and fluid in the original configuration are labeled as $\rho_{0}^{s}$ and $\rho_{0}^{f}$, respectively. Also recall that the Lagrangian total density of the material before growth is given by

$$\begin{array}{c} \rho_{0,\text{init}}=\rho_{0,\text{init}}^{s}+\rho_{0,\text{init}}^{f}=\left(1-n_{0}\right)\rho_{T}^{s}+n_{0}\rho_{T}^{f}, \end{array}$$ (27)

where $\rho_{0,\text{init}}^{s}$ is the Lagrangian apparent density of the solid before growth, and $\rho_{0,\text{init}}^{f}$ is the Lagrangian apparent density of the fluid before growth. Then we may write the grown density from Eq (26) as

$$\begin{array}{c} \rho_{0}=\rho_{0}^{s}+\rho_{0}^{f}=\left(1-n_{0}\right)\rho_{T}^{s}+n_{0}\rho_{T}^{f}+\int_{t_{0}}^{t}R_{0}^{s}d\bar{t}+\int_{t_{0}}^{t}R_{0}^{f}d\bar{t}, \end{array}$$ (28)

by separating out solid and fluid source terms.

We consider the solid and the fluid separately. For the solid, the grown apparent density in the original configuration is given by

$$\begin{array}{c} \rho_{0}^{s}=\left(1-n_{0}\right)\rho_{T}^{s}+\int_{t_{0}}^{t}R_{0}^{s}d\bar{t}. \end{array}$$ (29)

Here, we assume density preservation of the apparent density of the solid ${\hat{\rho }}_{0}^{s}=\rho_{0,\text{init}}^{s}$, which is a constant. With this assumption, $\dot{{\hat{\rho }}_{0}^{s}}=0$ and the time derivative of Eq (29) simplifies from Eq (14) to become

$$\begin{array}{c} {\dot{\rho }}_{0}^{s}={\dot{J}}^{g}\rho_{0,\text{init}}^{s}=R_{0}^{s}. \end{array}$$ (30)

Then following a similar derivation to the solid-only growth above, we obtain the solid source term

$$\begin{array}{c} R_{0}^{s}=\left(\vartheta^{3}\right)\left(3\dot{\vartheta }/\vartheta \right)\rho_{0,\text{init}}^{s}=3\rho_{0,\text{init}}^{s}\vartheta^{2}\dot{\vartheta }. \end{array}$$ (31)

Using the definition for initial apparent solid density, the solid source may be written as

$$\begin{array}{c} R_{0}^{s}=3\rho_{0}^{s^{*}}\vartheta^{2}\dot{\vartheta }=3\left(1-n_{0}\right)\rho_{T}^{s}\vartheta^{2}\dot{\vartheta }, \end{array}$$ (32)

where $\rho_{0}^{s^{*}}=\left(1-n_{0}\right)\rho_{T}^{s}$ is the solid density preserved by growth.

For the grown fluid density, we may similarly write

$$\begin{array}{c} \rho_{0}^{f}=n_{0}\rho_{T}^{f}+\int_{t_{0}}^{t}R_{0}^{f}d\bar{t}. \end{array}$$ (33)

Taking the time derivative, the growth rate of the apparent fluid density in the original configuration is given by

$$\begin{array}{c} {\dot{\rho }}_{0}^{f}=\dot{\overset{︵}{J^{g}{\hat{\rho }}_{0}^{f}}}={\dot{J}}^{g}{\hat{\rho }}_{0}^{f}+J^{g}\dot{{\hat{\rho }}_{0}^{f}}=R_{0}^{f}. \end{array}$$ (34)

With the assumptions of isotropic growth and density preservation of the apparent density of the fluid from the initial state to the intermediate configuration (i.e., ${\hat{\rho }}_{0}^{f}=\rho_{0,\text{init}}^{f}=n_{0}\rho_{T}^{f}$, which is a constant), Eq (34) becomes

$$\begin{array}{c} R_{0}^{f}={\dot{J}}^{g}\rho_{0,\text{init}}^{f}=3\rho_{0}^{f^{*}}\vartheta^{2}\dot{\vartheta }=3n_{0}\rho_{T}^{f}\vartheta^{2}\dot{\vartheta }, \end{array}$$ (35)

where $\rho_{0}^{f^{*}}=n_{0}\rho_{T}^{f}$ is the fluid density preserved by growth.

In summary, for isotropic growth and density preservation of the solid and the fluid, the source terms may be written as

$$\begin{array}{c} R_{0}^{s}=3\left(1-n_{0}\right)\rho_{T}^{s}\vartheta^{2}\dot{\vartheta },\;R_{0}^{f}=3n_{0}\rho_{T}^{f}\vartheta^{2}\dot{\vartheta }. \end{array}$$ (36)

Integrating the source terms from *t*_0_ to *t*, multiplying by the reference volume, and assuming that *ϑ*(*t*_0_) = 1, we obtain the added masses of the solid and fluid from growth:

$$\begin{array}{c} dm^{s,g}=\int_{t_{0}}^{t}R_{0}^{s}d\bar{t}dV_{0}=\rho_{T}^{s}\underset{\text{solid}\;\text{volume}\;\text{added}\;\text{by}\;\text{growth}}{\underset{︸}{\left(1-n_{0}\right)\left(\vartheta^{3}-1\right)dV_{0}}}, \end{array}$$ (37)

$$\begin{array}{c} dm^{f,g}=\int_{t_{0}}^{t}R_{0}^{f}d\bar{t}dV_{0}=\rho_{T}^{f}\underset{\text{fluid}\;\text{volume}\;\text{added}\;\text{by}\;\text{growth}}{\underset{︸}{n_{0}\left(\vartheta^{3}-1\right)dV_{0}}}. \end{array}$$ (38)

Recall that the final mass of the fluid after deformation may differ from the mass of the fluid after growth because elastic deformation can cause fluid flux.

Adding the fluid and solid sources together, the total source in Lagrangian coordinates is given by

$$\begin{array}{c} R_{0}=R_{0}^{s}+R_{0}^{f}=3[\left(1-n_{0}\right)\rho_{T}^{s}+n_{0}\rho_{T}^{f}]\vartheta^{2}\dot{\vartheta }=3[\rho_{0,\text{init}}]\vartheta^{2}\dot{\vartheta }, \end{array}$$ (39)

so growth preserves the initial density of the porous material, where *ρ*_0,init_ = (*dM*^*s*^ + *dM*^*f*^)/*dV*_0_ from Eq (27).

Further note that the sum of the density-specific source terms is given by

$$\begin{array}{c} \frac{R_{0}^{s}}{\rho_{T}^{s}}+\frac{R_{0}^{f}}{\rho_{T}^{f}}=3[\left(1-n_{0}\right)+n_{0}]\vartheta^{2}\dot{\vartheta }=3\vartheta^{2}\dot{\vartheta }. \end{array}$$ (40)

This sum of the normalized source terms will be appear in the fluid conservation equation and is the same as the density specific term appearing in solid-only growth, as in Eq (24).

#### Calculation of final porosity after growth and deformation

Independently from the type of growth considered, the mass of the grown solid may be written in Lagrangian coordinates as the initial solid mass plus a growth term, as in Eq (8). The initial solid mass may be written as the initial apparent density multiplied by the original volume:

$$\begin{array}{c} dM^{s}=\rho_{0,\text{init}}^{s}dV_{0}=\rho_{T}^{s}\left(1-n_{0}\right)dV_{0}. \end{array}$$ (41)

For isotropic growth at constant solid density $\rho_{0}^{s^{*}}$, the grown mass element of the solid is given by explicitly integrating the source term from Eq (8). Then also writing *dM*^*s*^ as in Eq (41), the expression for the grown mass may be written as

$$\begin{array}{c} dm^{s}=\rho_{T}^{s}\left(1-n_{0}\right)dV_{0}+\rho_{0}^{s^{*}}\left(\vartheta^{3}-1\right)dV_{0}. \end{array}$$ (42)

Note that if there is no growth, then *ϑ* = 1 and the masses are the same: *dm*^*s*^ = *dM*^*s*^. Equivalently to Eq (42), the grown mass element may be calculated in Eulerian coordinates by multiplying the new apparent density of the solid by the deformed volume *dV*. Then

$$\begin{array}{c} dm^{s}=\rho^{s}dV=\rho_{T}^{s}\left(1-n\right)dV=\rho_{T}^{s}\left(1-n\right)JdV_{0}, \end{array}$$ (43)

where both the definition of grown apparent density of the solid $\rho^{s}=\rho_{T}^{s}\left(1-n\right)$ and the relationship *dV* = *JdV*_0_ have been applied.

Setting Eqs (42) and (43) equal and then solving for the Eulerian porosity *n*, we obtain

$$\begin{array}{c} n=\underset{\text{mechanical}}{\underset{︸}{1-J^{-1}\left(1-n_{0}\right)}}-\underset{\text{growth}}{\underset{︸}{J^{-1}[\frac{\rho_{0}^{s^{*}}}{\rho_{T}^{s}}\left(\vartheta^{3}-1\right)]}}. \end{array}$$ (44)

Comparing this with the non-growth definition of the porosity *n* = 1 − *J*^−1^(1 − *n*_0_), we observe that this expression represents both a mechanical change and a growth contribution.

The specific form of Eq (44) depends on the choice of constant growth density $\rho_{0}^{s^{*}}$. The two obvious choices are that (1) the true density of the solid is preserved (so solid mass is added at the true density of the solid), and (2) the initial apparent density of the solid is preserved (so solid mass is added at the original apparent density of the solid while the fluid mass is simultaneously added at the original apparent density of the fluid). Note that we may equivalently calculate the final porosity via the final volume of the fluid. For further details of this calculation, see Armstrong [41].

#### Porosity for solid-only growth

For growth at the true density of the solid, $\rho_{0}^{s^{*}}=\rho_{T}^{s},$ the grown mass of the solid is given by

$$\begin{array}{c} dm^{s}=\rho_{T}^{s}\left(\vartheta^{3}-n_{0}\right)dV_{0}. \end{array}$$ (45)

The porosity after growth and deformation simplifies from Eq (44) to

$$\begin{array}{c} n=1-J^{-1}\left(\vartheta^{3}-n_{0}\right). \end{array}$$ (46)

If we additionally assume no volumetric change from elastic deformation, i.e. *J*^*e*^ = 1 so *J* = *J*^*e*^ *J*^*g*^ = *ϑ*^3^, then the porosity becomes

$$\begin{array}{c} n=1-\vartheta^{-3}\left(\vartheta^{3}-n_{0}\right)=\vartheta^{-3}n_{0}. \end{array}$$ (47)

So for solid-only growth that preserves density, growth changes the porosity. This occurs because adding only solid material will change the ratio of solid to fluid. Without elastic deformation, the porosity would only be the same as the initial porosity with the additional assumption of *J*^*g*^ = 1, i.e. no volumetric change from growth.

#### Porosity for solid and fluid growth

For growth at the apparent density of the solid, the solid growth density is given by $\rho_{0}^{s^{*}}=\left(1-n_{0}\right)\rho_{T}^{s}$. The grown mass of the fluid (before flux caused by elastic deformation) is given by

$$\begin{array}{c} dM^{f}+dm^{f,g}=\rho_{T}^{f}n_{0}dV_{0}+\rho_{T}^{f}n_{0}\left(\vartheta^{3}-1\right)dV_{0}=\rho_{T}^{f}n_{0}\vartheta^{3}dV_{0}. \end{array}$$ (48)

The grown mass of the solid is given by

$$\begin{array}{c} dm^{s}=\rho_{T}^{s}\left(1-n_{0}\right)\vartheta^{3}dV_{0}. \end{array}$$ (49)

and Eq (44) simplifies to

$$\begin{array}{c} n=1-J^{-1}\left(1-n_{0}\right)\vartheta^{3}. \end{array}$$ (50)

Note that if we assume no volumetric change from elastic deformation, *J*^*e*^ = 1 so *J* = *J*^*e*^ *J*^*g*^ = *ϑ*^3^ and the porosity becomes

$$\begin{array}{c} n=1-\vartheta^{-3}\left(1-n_{0}\right)\vartheta^{3}=n_{0}. \end{array}$$ (51)

Thus, this case (growth that preserves the apparent densities of the solid and the fluid) represents adding additional porous media with the same porosity.

### Porosity constraint on the GMPHETS model

Without further restrictions, some growth models may cause unbounded growth (e.g., the simplistic time-dependent growth law $\dot{\vartheta }=\alpha$). In particular, the unbounded growth may be such that it invalidates the behavior of the model. For admissibility, the theory of porous media requires a porosity of 0 ≤ *n* ≤ 1. In particular, GMPHETS models the interaction between solid, fluid, and species. If the material becomes either completely fluid (*n* = 1) or completely solid (*n* = 0), the GMPHETS porous media model is no longer valid.

For the system to be valid for GMPHETS, the porosity (from Eq (44)) must be such that

$$\begin{array}{c} 0<1-J^{-1}[\left(1-n_{0}\right)+{\bar{\rho }}^{s}(\vartheta^{3}-1)]<1, \end{array}$$ (52)

where ${\bar{\rho }}^{s}=\rho_{0}^{s^{*}}/\rho_{T}^{s}$ is the normalized solid growth density. Then equivalently, we require that

$$\begin{array}{c} \sqrt[{3}]{1+\frac{1}{{\bar{\rho }}^{s}}\left(J-1+n_{0}\right)}>\vartheta >\sqrt[{3}]{1+\frac{1}{{\bar{\rho }}^{s}}\left(n_{0}-1\right)}. \end{array}$$ (53)

Once the model reaches either limit, the porous media model is no longer valid because the material is either purely solid or purely fluid. One approach to handle this invalidity would be to include some sort of transitional behavior between a fluid-only model, a porous media model, and a solid model, but that is beyond the scope of this paper. For the work presented here, we prevent the material from reaching this critical stretch by either restricting growth time or including a nonlinear growth parameter that bounds *ϑ* (introduced by Lubarda and Hoger [33] and detailed in Eq (76)).

### GMPHETS conservation and constitutive equations

For a derivation of the MPHETS conservation equations with growth, we refer the reader to S2 Appendix. We summarize the Lagrangian conservation equations here. Firstly, linear momentum is conserved:

$$\begin{array}{c} \frac{\partial \left[F_{ip}S_{pj}\right]}{\partial X_{i}}=0,\;\nabla \cdot \left(FS\right)=0, \end{array}$$ (54)

for deformation gradient *F*_*ip*_, second Piola-Kirchhoff stress tensor *S*_*pj*_ and undeformed configuration *X*_*i*_. A combination of the conservation of mass of the solid, conservation of the mass of the fluid, and incompressibility of each constituent, becomes

$$\begin{array}{c} \frac{\partial {\tilde{j}}_{k}^{fr}}{\partial X_{k}}+JH_{ij}{\dot{E}}_{ij}-3\vartheta^{2}\dot{\vartheta }=0,\;\nabla \cdot j^{fr}+JH:\dot{E}-3\vartheta^{2}\dot{\vartheta }=0; \end{array}$$ (55)

for relative fluid flux ${\tilde{j}}_{k}^{fr}$; Jacobian *J* = det(*F*_*ij*_); Finger’s strain tensor $H_{ij}=F_{ip}^{-1}F_{jp}^{-1}$; Green strain $E_{ij}=\frac{1}{2}(F_{pi}F_{pj}-\delta_{ij})$ for *δ*_*ij*_ the identity tensor; and *ϑ* the growth stretch multiplier. Eq (55) will henceforward referred to as the fluid conservation equation.

Finally, the mass of the species is conserved:

$$\begin{array}{c} \frac{\partial {\tilde{j}}_{k}^{cr}}{\partial X_{k}}+JH_{ij}{\dot{E}}_{ij}c-3{\bar{\rho }}^{s}\vartheta^{2}\dot{\vartheta }c+Jn\dot{c}=0,\;\nabla \cdot j^{cr}+JH:\dot{E}c-3{\bar{\rho }}^{s}\vartheta^{2}\dot{\vartheta }c+Jn\dot{c}=0, \end{array}$$ (56)

for relative species flux ${\tilde{j}}_{k}^{cr}$, concentration *c*, normalized solid growth density ${\bar{\rho }}^{s}=\rho_{0}^{s^{*}}/\rho_{T}^{s}$, and porosity *n* such that

$$\begin{array}{c} n=1-J^{-1}[\left(1-n_{0}\right)+{\bar{\rho }}^{s}\left(\vartheta^{3}-1\right)], \end{array}$$ (57)

where *n*_0_ is the initial porosity of the material. The conservation of linear momentum Eq (54) appears the same as in traditional MPHETS theory, but the stress will be modified by growth. Growth adds terms that appear directly in the conservation equations for the fluid Eq (55) and species Eq (56); these terms are not present in traditional MPHETS theory and also were not considered in [40]. In the fluid equation, growth is a purely volumetric term; for the two cases discussed—growth at constant solid density (solid-only growth at $\rho_{T}^{s}$) and both solid and fluid growth at constant apparent density (solid growth at $\rho_{T}^{s}\left(1-n_{0}\right)$ and fluid growth at $\rho_{T}^{f}n_{0}$)—the volumetric term is identical. The species equation depends explicitly on the porosity, and so differs with the particular growth case considered. Note that the porosity is not assumed to be constant during growth, which represents a relaxation of the assumptions from [40].

#### Effective stress and the growth pullback

The effective stress is also modified by growth. For example, consider a growing cube of a compressible solid material with a fixed boundary. As the material grows and adds mass, elastic stresses compress the material back to the original configuration. In general, standard growth theory assumes that growth occurs to bring the system to some new fictitious, stress-free configuration that may be incompatible (as in Fig 1). Then the elastic deformation brings the system back to a compatible configuration. So residual elastic stresses may develop even if the boundary conditions are not fixed.

Thus, in addition to the effective stress principle splitting the stress into stress on the fluid and the effective stress, we must also consider growth. So stress becomes a function of total deformation and growth, **S**(**F**,**F**^*g*^).

The growth pullback into the undeformed configuration is given by

$$\begin{array}{c} S^{\text{eff}}=F^{g-1}S^{\text{eff},e}F^{g-T}, \end{array}$$ (58)

[17]. For isotropic growth the effective stress then becomes

$$\begin{array}{c} S^{\text{eff}}=F^{g-1}S^{\text{eff},e}F^{g-T}=\frac{1}{\vartheta^{2}}S^{\text{eff},e},\;\text{where}\;S^{\text{eff},e}=\frac{\partial W^{\text{eff}}}{\partial E^{e}}, \end{array}$$ (59)

for free energy *W*^eff^. Note that the elastic portion of the effective stress **S**^eff,*e*^ is based on the elastic strain only. This elastic strain changes with growth because during a growth step, **F** is held fixed; thus any changes in the growth deformation **F**^*g*^ cause corresponding changes in the elastic deformation **F**^*e*^. Hence, growth causes a change in the stress. Combining the effective stress principle with the growth pullback, the second Piola-Kirchhoff stress is given for isotropic growth as

$$\begin{array}{c} S\left(F,\vartheta ,W^{\text{eff}},p^{f}\right)=F^{g-1}S^{\text{eff},e}F^{g-T}-JHp^{f}=\frac{1}{\vartheta^{2}}S^{\text{eff},e}-JHp^{f} \end{array}$$ (60)

#### Other constitutive equations

The other constitutive equations are analogous to those without growth, detailed in [3, 15], for example. For completeness, they are listed below. Hyperelasticity defines the effective stress in terms of the free energy *W*^eff^ as

$$\begin{array}{c} S_{ij}^{\text{eff},e}=\frac{\partial W^{\text{eff}}}{\partial E_{ij}^{e}},\;S^{\text{eff},e}=\frac{\partial W^{\text{eff}}}{\partial E^{e}}. \end{array}$$ (61)

The effective stress principle splits the stress into the pore fluid pressure and the remaining ‘effective’ stress, where

$$\begin{array}{c} S_{ij}=S_{ij}^{\text{eff}}-JH_{ij}p^{f},\;S=S^{\text{eff}}-JHp^{f}. \end{array}$$ (62)

The Onsager equations are a generalized version Darcy’s and Fick’s laws, and couple the species and the pore fluid pressure. For ${\tilde{j}}_{i}^{fr}$ the Lagrangian relative fluid flux and ${\tilde{j}}_{i}^{cr}$ the Lagrangian relative species flux,

$$\begin{array}{c} {\tilde{j}}_{i}^{fr}=-{\tilde{L}}_{ij}^{ff}\frac{\partial {\tilde{\mu }}^{f^{*}}}{\partial X_{j}}-{\tilde{L}}_{ij}^{fc}\frac{\partial {\tilde{\mu }}^{c^{*}}}{\partial X_{j}},\;{\tilde{j}}^{fr}=-{\tilde{L}}^{ff}\frac{\partial {\tilde{\mu }}^{f^{*}}}{\partial X}-{\tilde{L}}^{fc}\frac{\partial {\tilde{\mu }}^{c^{*}}}{\partial X}, \end{array}$$ (63)

$$\begin{array}{c} {\tilde{j}}_{i}^{cr}=-{\tilde{L}}_{ij}^{cf}\frac{\partial {\tilde{\mu }}^{f^{*}}}{\partial X_{j}}-{\tilde{L}}_{ij}^{cc}\frac{\partial {\tilde{\mu }}^{c^{*}}}{\partial X_{j}},\;{\tilde{j}}^{cr}=-{\tilde{L}}^{cf}\frac{\partial {\tilde{\mu }}^{f^{*}}}{\partial X}-{\tilde{L}}^{cc}\frac{\partial {\tilde{\mu }}^{c^{*}}}{\partial X}, \end{array}$$ (64)

where ${\tilde{L}}_{ij}^{ff},{\tilde{L}}_{ij}^{fc},{\tilde{L}}_{ij}^{cf},{\tilde{L}}_{ij}^{cc}$ are given material parameters that can be calculated from the isotropic Eulerian MPHETS parameters of porosity *k*^*ff*^, convection coupling coefficient *b*^*fc*^ = *b*^*cf*^, and diffusivity *d*^*cc*^. The Lagrangian form of the ${\tilde{L}}_{ij}$ parameters (with the assumption of isotropic Eulerian Darcy/Fick parameters) are given in [40] as

$$\begin{array}{c} {\tilde{L}}_{ij}^{ff}=JH_{ij}k^{ff}, \end{array}$$ (65)

$$\begin{array}{c} {\tilde{L}}_{ij}^{fc}=JH_{ij}(k^{ff}b^{fc}c)={\tilde{L}}_{ij}^{cf}, \end{array}$$ (66)

$$\begin{array}{c} {\tilde{L}}_{ij}^{cc}=JH_{ij}(\frac{c}{\bar{R}\theta }d^{cc}+cb^{cf}k^{ff}b^{fc}c), \end{array}$$ (67)

for universal gas constant $\bar{R}=8.31\;\left(J/K/mol\right)$ and material temperature *θ* (assumed to be constant at 310 *K*).

The primary and secondary variables are linked by the standard mechano-chemical potentials:

$$\begin{array}{c} {\tilde{\mu }}^{f^{*}}=p^{f}+p_{0}^{o}-R\theta \phi^{c}c, \end{array}$$ (68)

where $p_{0}^{o}$ is a baseline osmotic potential, *R* is the universal gas constant, *θ* is the temperature (assumed to be constant) and *ϕ*^*c*^ is the osmotic coefficient of the species in the material, and

$$\begin{array}{c} {\tilde{\mu }}^{c^{*}}=\mu_{o}^{c}+R\theta \log\left(\gamma_{\text{mat}}^{c}c\right), \end{array}$$ (69)

where $\mu_{o}^{c}$ is a baseline chemical potential and $\gamma_{\text{mat}}^{c}$ is the activity coefficient of the species in the material [3, 40].

In summary, the structural model is based on material displacement (Eq (54)). The displacement is coupled to the pore fluid pressure through the effective stress principle (Eq (60)). Finally, the pore fluid pressure and chemical concentration are coupled through the Onsager Eqs (63) and (64), which provides a fully coupled system.

### Material law for effective stress

To illustrate the newly developed model, we choose an isotropic Neo-Hookean form used by Göktepe et al. [6], where for material parameters *λ*, *μ*,

$$\begin{array}{c} W^{\text{eff}}\left(I_{1},J^{e}\right)=\frac{1}{2}\lambda \ln^{2}\left(J^{e}\right)+\frac{\mu }{2}[I_{1}-3-2\ln\left(J^{e}\right)]. \end{array}$$ (70)

Note that by definition, *C*_10_ = *μ*/2, *D*_1_ = 2/*κ*, and $\lambda =\kappa -\frac{2}{3}\mu$, so the parameter set {*λ*, *μ*} may be calculated from the parameter set {*C*_10_, *D*_1_}.

The effective, elastic second Piola-Kirchhoff stress is then given by

$$\begin{array}{c} S^{\text{eff},e}=2\frac{\partial W^{\text{eff}}}{\partial C^{e}}=\left(\lambda \ln\left(J^{e}\right)-\mu \right)C^{e-1}+\mu I, \end{array}$$ (71)

and the elastic Lagrangian tangent modulus is given as

$$\begin{array}{c} L_{ijkl}^{e}=2\frac{\partial S_{ij}^{\text{eff},e}}{\partial C_{kl}^{e}}=\lambda C_{ij}^{e-1}C_{kl}^{e-1}+\left(\mu -\lambda \ln\left(J^{e}\right)\right)(C_{ik}^{e-1}C_{lj}^{e-1}+C_{il}^{e-1}C_{kj}^{e-1}). \end{array}$$ (72)

This constitutive model will be used in the finite element representation of the growing MPHETS model.

### Finite element theory for a growing MPHETS material

In this section we introduce the finite element formulation for the growing MPHETS model. GMPHETS theory was implemented as an in-house finite element code, programmed in MATLAB. The elements are three-noded, linear axisymmetric triangles, where primary variables (displacement, fluid potential, and chemical potential) are defined at the nodes, and secondary variables (pore fluid pressure and concentration) are defined at the Gauss points. The primary variables are interpolated linearly and are continuous, while the secondary variables are piecewise constant. Though this is a low-order interpolation, our finite element implementation is sufficient to model a GMPHETS material for a dense mesh, and demonstrates the potential of the GMPHETS theory. For integration on the axisymmetric triangles, we use Gauss Quadrature evaluated at the single Gauss point. For additional background on finite elements, we refer the reader to [45–47].

#### Choice of solution variables

The partition coefficient, or the ratio of activity coefficients on either side of a material interface, defines the relative concentration on either side material boundaries. In general, differing activity coefficients result in a concentration discontinuity at these locations. Because the concentration is not continuous, the osmotic pressure is also not continuous, and hence, the pore fluid pressure is not continuous. Thus, the concentration and pore fluid pressure are not suitable as primary variables, which must be continuous on the nodes. To handle this discontinuity, one may wish to use a penalty method [48] or secondary variables [3, 14, 40]. In this work, we use the latter approach. The classical mechano-chemical potentials are defined above in Eqs (68) and (69).

The total pressure is defined in Eq (68) as the sum of the pore fluid pressure and the osmotic pressure; note that the osmotic pressure is the sum of a datum based on the lowest concentration expected in the problem and the osmotic pressure expressed by the current chemical concentration. In Eq (69), the quantity $\gamma_{i}^{c}c_{i}$ represents the activity *a*_*i*_ of the chemical species. The term $\mu_{0}^{c}$ in the chemical potential is a datum based upon the lowest concentration expected in the problem and ensures that the chemical species concentration remains above zero; thus the logarithm in the chemical potential is well-defined. The GMPHETS code contains both primary variables (defined at the nodes) and secondary variables (defined at the Gauss points). The standard mechano-chemical potentials relate the primary variables to the secondary variables, as in [40]. This allows the finite element equations to be formulated as a function of the primary variables; the secondary variables are then carried along as internal variables.

### Numerical algorithm

#### Algorithm pseudocode

**Fig 2:**
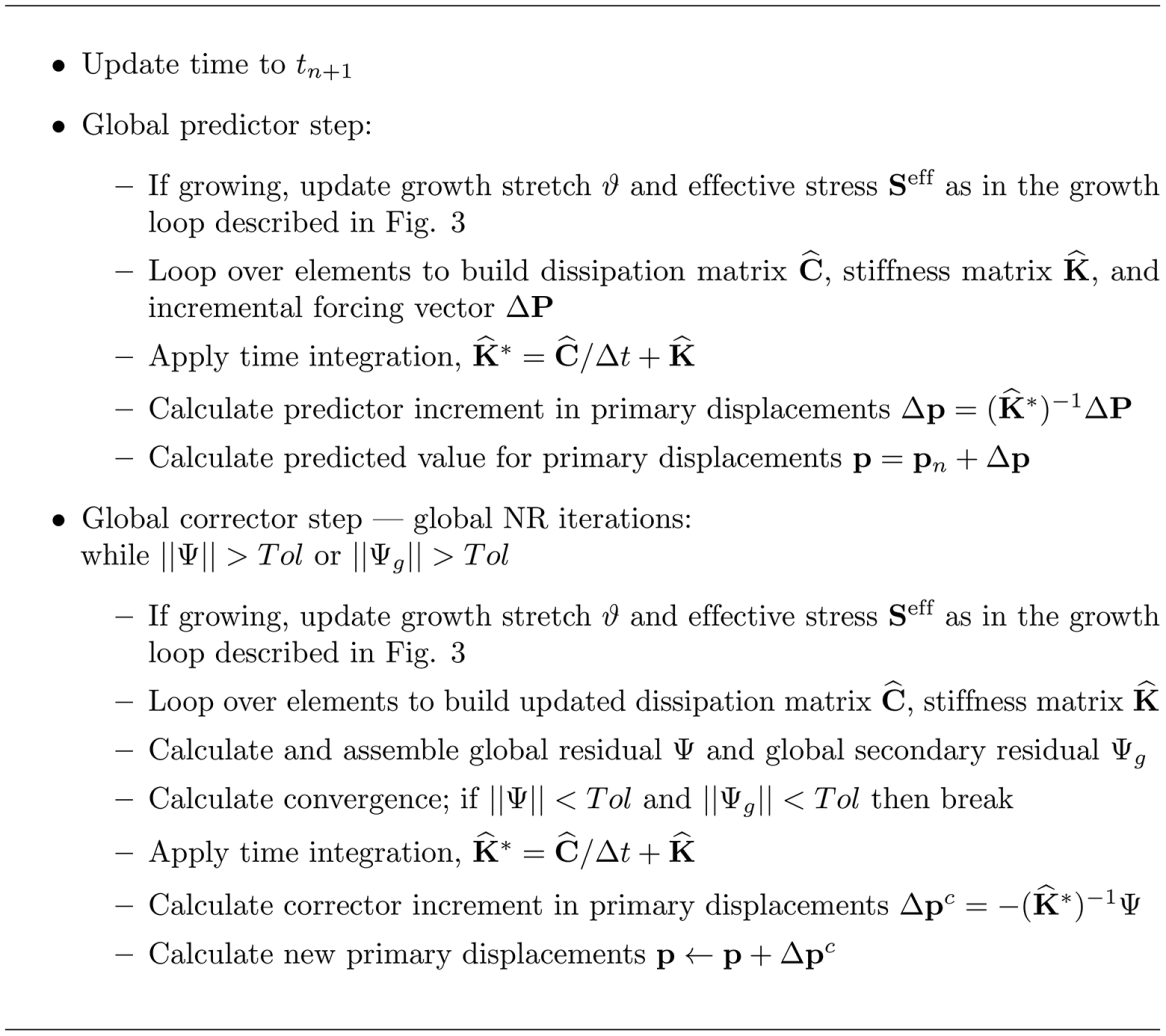
Finite element pseudocode for GMPHETS. Variables without subscripts are evaluated at the current time step and iteration. $\hat{C}$ is the global dissipation matrix, $\hat{K}$ is the global stiffness matrix, **p** is the vector of primary displacements, Ψ is the primary residual, and Ψ_*g*_ is the secondary residual.

**Fig 3:**
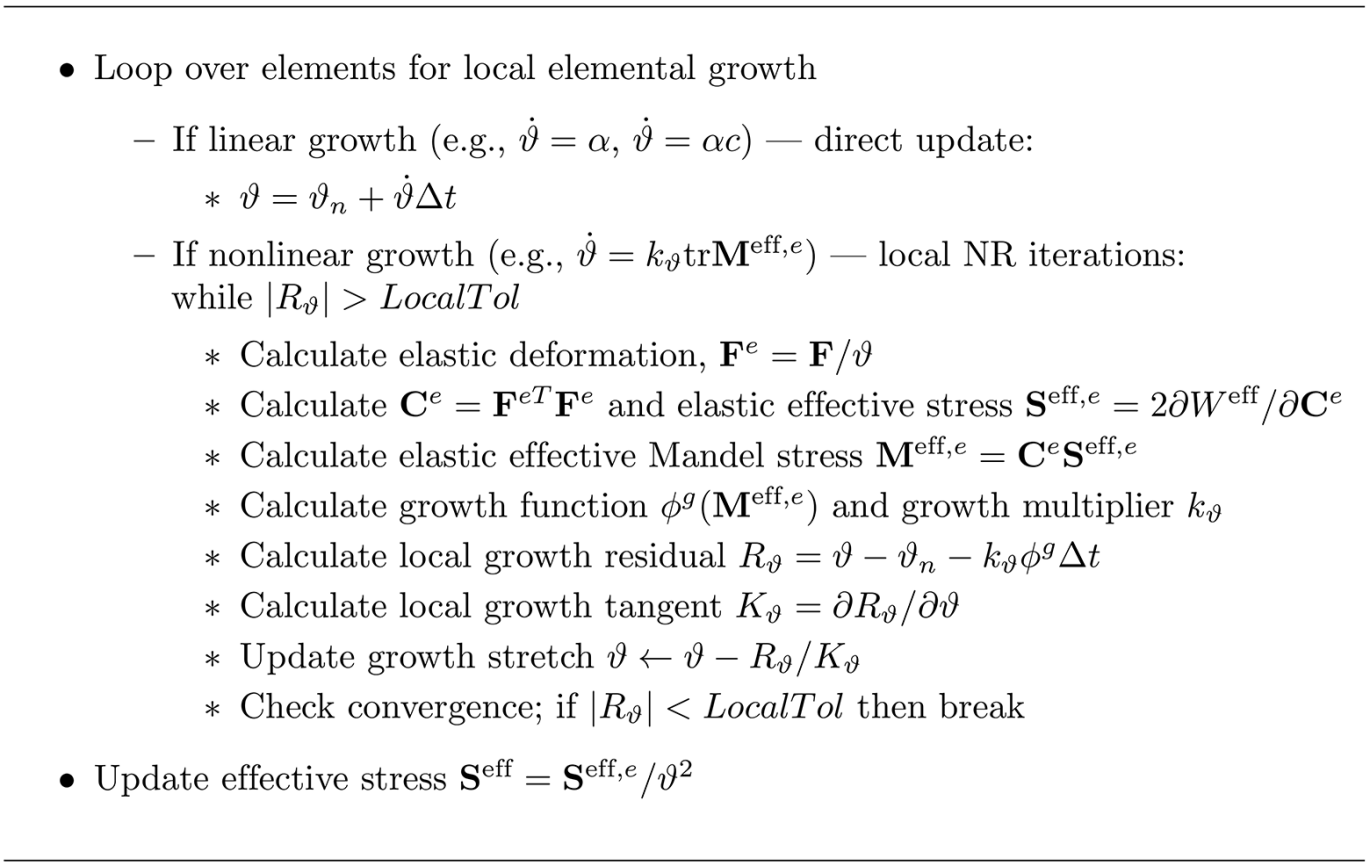
Local element update for GMPHETS with isotropic growth. The growth update algorithm is modified for GMPHETS theory from [6]. Variables without subscripts are evaluated at the current time step and iteration. For the linear case, growth is updated directly. For the nonlinear case, backward Euler is used for stability.

The finite element code is run with a traditional Newton-Raphson (NR) predictor corrector method. The full algorithm used is detailed in Fig 2. Growth has been implemented following the algorithm from [6] (adapted for GMPHETS and detailed in Fig 3).

The full finite element algorithm for nonlinear growth contains a nested set of two Newton-Raphson iterations. For a given time step, local growth is iterated with Newton-Raphson on each element—given a fixed **F**, the nonlinear growth stretch is iterated until convergence. Then, using the updated elemental growth stretch *ϑ*, the new stress is calculated and a single global Newton-Raphson iteration takes place. Thus, local growth iterations are run for each element during every global iteration.

To solve the global conservation equations, the algorithm employs a traditional predictor corrector method to handle nonlinearity, pairing a step of forward Euler with corrections using backward Euler time integration for stability. A backward Euler algorithm means that the current value of all variables is used during the correction step. To obtain a prediction for the current time step, the incremental global finite element equations are linearized to obtain stiffness matrix $\hat{K}$, dissipation matrix $\hat{C}$, and incremental loading vector Δ**P**. After applying the time integrator, the equations are then solved to obtain Δ**p**; the predicted value of the primary displacements is then given by **p**_*n*+1_ = **p**_*n*_ + Δ**p**. This predictor is then fed into the Backward Euler algorithm until either the primary and secondary residuals (given by Ψ, Ψ_*g*_, respectively) have converged; or the primary and secondary variables have converged; or the maximum number of iterations has been exceeded (which usually indicates that the time step is too large). A number of growth laws will be considered. For a nonlinear update, local Newton-Raphson iterations on each element update the growth stretch.

#### Local Newton-Raphson iterations to update growth stretch

If the growth rate function chosen is nonlinear, we follow [6, 17] and use local Newton-Raphson iterations on each element to update the growth stretch. For details of how to calculate such local Newton-Raphson tangents and residuals, we refer the reader to [6].

Briefly, while the growth **F**^*g*^ is being updated, total deformation **F** is by assumption fixed. Thus, all displacement values are fixed. The local Newton-Raphson iterations require a local growth residual *R*_*ϑ*_ and a local growth tangent modulus *K*_*ϑ*_ for each of the nonlinear growth cases following. Due to space considerations, the calculation of local Newton-Raphson iterations is omitted. For details, see Armstrong [41].

#### Growth laws

Each of the growth update functions considered is summarized below.

*Linear time-driven growth*. The simplest growth function considered is

$$\begin{array}{c} \dot{\vartheta }=\alpha , \end{array}$$ (73)

for constant growth parameter *α*. Then growth is independent of time and space. This simple linear growth may be updated via the finite difference expression

$$\begin{array}{c} \vartheta_{n+1}=\vartheta_{n}+\alpha \Delta t. \end{array}$$ (74)

*Nonlinear concentration-driven growth* Suppose that we have a more complicated function for concentration driven growth, where *ϕ*^*g*^(*c*) is some function of concentration, and both growth and resorption are governed by nonlinear parameter *k*_*ϑ*_. Let the time evolution of the growth stretch be defined as

$$\begin{array}{c} \dot{\vartheta }=k_{\vartheta }\left(\vartheta \right)\phi^{g}\left(c\right),\;\text{where}\;\phi^{g}\left(c\right)=c-c_{\text{thresh}}, \end{array}$$ (75)

for concentration threshold *c*_thresh_. The nonlinear parameter *k*_*ϑ*_ is akin to the growth stretch coefficient used in [6, 17], originally introduced by [33] to prevent unlimited growth. It is defined in terms of parameters *τ*^±^, *ϑ*^max^, *ϑ*^min^, *γ*^±^ as

$$\begin{array}{c} k_{\vartheta }\left(\vartheta \right)=\{\begin{array}{c} \frac{1}{\tau^{+}}{\left(\frac{\vartheta^{\text{max}}-\vartheta }{\vartheta^{\text{max}}-1}\right)}^{\gamma^{+}},\;\text{for}\;\phi^{g}>0\\ \frac{1}{\tau^{-}}{\left(\frac{\vartheta -\vartheta^{\text{min}}}{1-\vartheta^{\text{min}}}\right)}^{\gamma^{-}},\;\text{for}\;\phi^{g}<0 \end{array}. \end{array}$$ (76)

The material parameters *ϑ*^max^ and *ϑ*^min^ govern the maximal and minimal stretch of the material, respectively, while parameters *τ*^±^, *γ*^±^ govern the relaxation rate—the time that it takes for the growth stretch to reach *ϑ*^max^ or *ϑ*^min^ [17].

*Nonlinear stress-driven growth* A material may respond to stress such that it grows when under tension and resorbs when under compression to minimize the stress in the material. Here, we adapt the approach presented by Himpel et al. [17] and Göktepe et al. [6] for a solid-only material. Because these authors consider a purely solid material, the material stress is equal to the effective stress [6, 17]. In contrast, our model contains an interstitial fluid, and thus has a pore fluid pressure. We assume that growth is a function of the effective stress.

Then, let the growth function be given as

$$\begin{array}{c} \dot{\vartheta }=k_{\vartheta }\left(\vartheta \right)\phi^{g}\left(M^{\text{eff},e}\right), \end{array}$$ (77)

where *k*_*ϑ*_(*ϑ*) is defined above in Eq (76) and *ϕ*^*g*^ is some function dependent on stress. Recall that the effective, elastic Mandel stress is given in Eq (2). The simplest version of Eq (77) is for *ϕ*^*g*^(**M**^eff,*e*^) = tr(**M**^eff,*e*^). The trace of the Mandel stress was chosen in [6, 17] because it is energy-conjugate to the growth velocity gradient in the intermediate configuration ${\hat{L}}^{g}$. Then the growth law may be written as

$$\begin{array}{c} \dot{\vartheta }=k_{\vartheta }\left(\vartheta \right)\text{tr}\left(M^{\text{eff},e}\right). \end{array}$$ (78)

#### Incremental tangent modulus for global Newton-Raphson iterations

The weak form of the conservation equations, used to calculate the primary residual, are located in S3 Appendix. Incremental forms of the conservation equations, which are used to assemble the global tangent modulus, are located in S4 Appendix.

## Results

To illustrate the capabilities of the GMPHETS model, we consider a few different growth laws applied to a Neo-Hookean material. For time-dependent growth, we compare a porohyperelastic (PHE) model of a cylinder run with either a solid-only source term or a combined solid/fluid source term, and match both simulations to a time-dependent analytical solution. For stress-dependent growth, we show that after growth the gradient of the effective hoop stress for an MPHETS model of an axisymmetric cylinder is lower than the gradient in hoop stress for a hyperelastic (HE) model. Finally, for concentration-dependent growth, we consider how changing the balance of permeability *k*^*ff*^, convection coefficient *b*^*cf*^ and diffusivity *d*^*cc*^ can affect the final growth outcome.

### Model geometry and parameters

Each problem simulates growth of an artery modeled as a plane-strain cylinder. The artery has an inner radius of 1 *mm*, an outer radius of 1.25 *mm*, and is internally pressurized to 100 *mmHg* with zero external pressure. Model geometry and material parameters are derived from experimental data of a porcine left anterior descending coronary artery [43, 49]; material parameters are located in Table 1. The permeability *k*^*ff*^, convection coupling coefficient *b*^*cf*^, and diffusivity *d*^*cc*^ cause the chemical species to distribute slowly across the mesh during the simulation.

**Table 1:** Material parameters used in test problems.

*C*_10_ [*Pa*]	*D*_1_ [*Pa*^−1^]	*k*^*ff*^ [*m*^4^/(*N* ⋅ *s*)]	*b*^*cf*^ [unitless]	*d*^*cc*^ [*m*^2^/*s*]	$\gamma_{\text{mat}}^{c}$	*ϕ*^*c*^ [unitless]	*n*_0_ [unitless]
1e6	5.5e-9	2e-14	6e-4	4.55e-14	0.5	0	0.5

Material parameters *C*_10_, *D*_1_, *k*^*ff*^, *b*^*cf*^, *d*^*cc*^ are adapted from [43, 49].

We assume the artery is sitting in a bath such that the internal bath has a concentration of 6.40*e* − 3 *mol*/*m*^3^, the external bath has a concentration of 6.40*e* − 4 *mol*/*m*^3^, and the partition coefficient for both baths is given as $\gamma_{\text{mat}}^{c}/\gamma_{\text{bath}}^{c}=0.5$. Via the partition coefficient, the concentration just inside the leftmost point of the cylinder is subject to a concentration of 0.0128 *mol*/*m*^3^, and the concentration just inside the external part of the cylinder is 1.28*e* − 3 *mol*/*m*^3^.

By assumption, the material has an initial porosity of *n*_0_ = 0.5, indicating equal parts by volume of solid and fluid. For simplicity, all growth examples presented in this paper assume that the osmotic coefficient *ϕ*^*c*^ is zero (i.e., there is no osmosis). Growth rate parameters {*α*, *τ*^±^, *γ*^±^} are chosen arbitrarily and illustrate the capabilities of the model; future work is needed to determine the time scale of growth for a specific physiological model. Growth may occur either simultaneously with or after consolidation. For the time-dependent growth problem, growth occurs after consolidation is complete, while in the stress- and concentration-driven growth problems, growth occurs simultaneously with fluid and species transport. Unless otherwise indicated, stress will be plotted for Gauss points averaged to the center node of adjacent triangles, following [50, 51].

### Time-dependent growth of a rigid PHE cylinder with solid vs. solid/fluid mass source

We first simulate time-dependent growth of a rigid, pressurized artery to compare against a time-dependent analytical solution. For the case of an internally pressurized, rigid porohyperelastic cylinder, we can calculate the analytical solution for the stresses and pore fluid pressure due to time-driven growth. Here, rigidity indicates that spatial displacements in the radial and axial directions are held fixed at every point; the pore fluid pressure is not fixed and is subject only to the boundary conditions at the edges of the material. The derivation of the analytical solution is presented in S5 Appendix.

For the finite element solution, a cylinder representing an artery is meshed as a strip of 120 elements. First, the cylinder consolidates to steady-state (which represents in-vivo status) and then a time-dependent growth law is applied (given by Eq (73) with *α* = 0.0008). For this test case we will consider two cases of growth: a solid-only mass source and a solid/fluid mass source that preserves the initial porosity during growth.

Recall that ${\bar{\rho }}^{s}$ is the constant, normalized solid growth density, defined as ${\bar{\rho }}^{s}=\rho_{0}^{s^{*}}/\rho_{T}^{s}$. For the solid-only material source, the true density of the solid is preserved during growth. Then $\rho_{0}^{s^{*}}=\rho_{T}^{s}$ and hence ${\bar{\rho }}^{s}=1$. For the combined solid/fluid mass source, growth preserves the initial apparent densities of the solid and the fluid (given by $\left[\left(1-n_{0}\right)\rho_{T}^{s}\right]$ and $\left[n_{0}\rho_{T}^{f}\right]$, respectively) which then preserve the initial porosity in the intermediate configuration during growth. The porosity in the deformed configuration will in general not be preserved due to fluid flux from elastic deformation. In this test problem, the final porosity will not match the initial porosity because the constraint of rigidity causes fluid flux. Using the solid growth density, for solid/fluid growth, the normalized solid growth density is given by ${\bar{\rho }}^{s}=\left(1-n_{0}\right)$. For an initial porosity of 0.5, this simplifies to ${\bar{\rho }}^{s}=0.5$. The growth parameters are listed in Table 2.

**Table 2:** Growth parameters for time-dependent growth in a rigid cylinder.

*α*	${\bar{\rho }}^{s}$
0.0008	0.5; 1

All growth parameters are unitless.

For either growth source, this simple time-dependent growth model is unbounded; as mentioned above, the amount of growth time is then restricted by the porosity. The most limiting case is for solid-only growth; for a combination solid/fluid material source, the amount of growth in a rigid solid could continue for a longer time period before becoming inadmissible. Using the limiting case and applying the simple time update of *ϑ* = *αt* + 1 to the growth restriction from Eq (53), one may calculate that the limiting time occurs at approximately 181 seconds of growth. Note that this limit is independent of time step size and depends only on total growth time and the value of *α*. Thus, after consolidation, we apply the growth law for 150 seconds (300 steps at *dt* = 0.5*s*) to not exceed the maximum amount of allowable growth.

For both cases of solid-only and solid/fluid source terms, many of the results remain the same (including displacement, pore fluid pressure, and stress). With the exception of the porosity, all figures will be plotted for the solid-only source case; figures from the solid/fluid case are identical.

**Fig 4:**
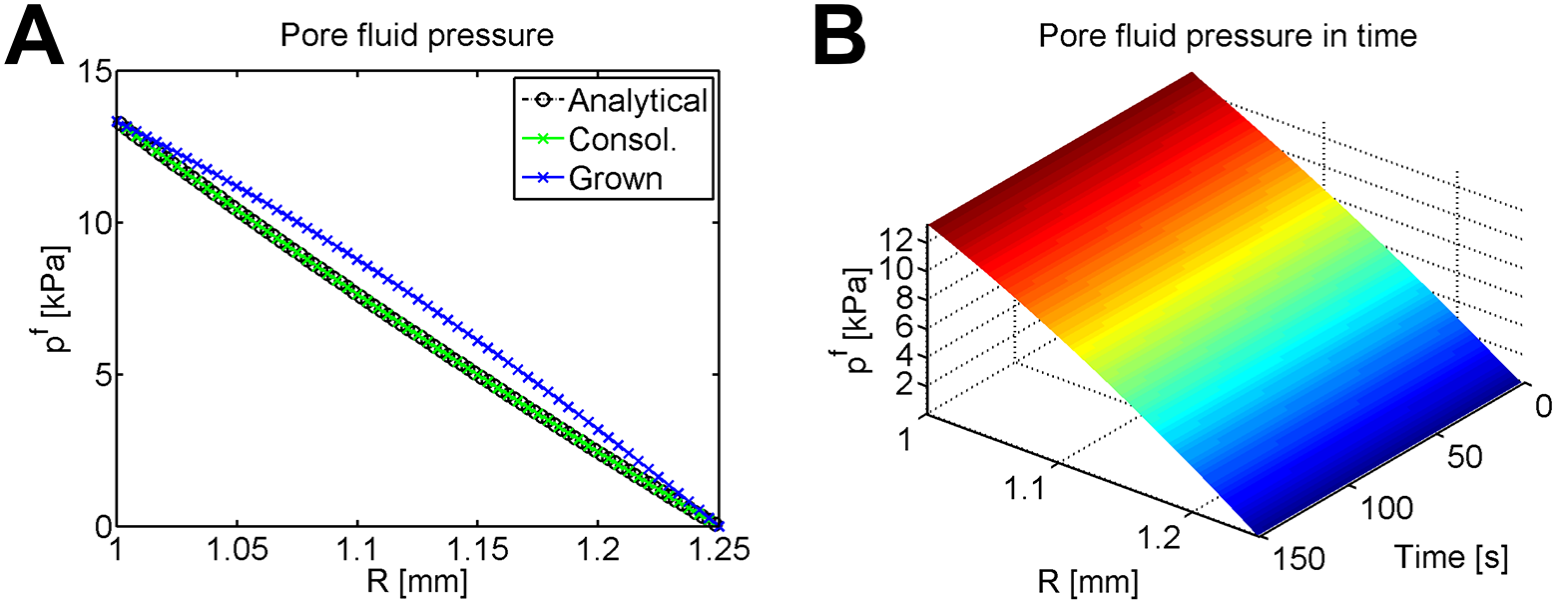
Pore fluid pressure for time-driven growth of an internally pressurized, rigid cylinder. (A) Consolidated and final pore fluid pressure compared with the analytical consolidated steady state for no growth, plotted at nodes. Recall that without osmosis, fluid potential is equal to pore fluid pressure. (B) Evolution of pore fluid pressure during growth, plotted at Gauss points.

The pore fluid pressure reaches the consolidated state immediately due to the restriction of a rigid material. The consolidated state matches the analytical steady solution for fluid pressure in a rigid cylinder without growth, shown in Fig 4A; the consolidated state (in green) lies directly on top of the analytical value (plotted in black). Rapid growth causes a shift rightwards in the fluid pressure (shown in blue); the fluid pressure after growth is higher than the consolidated steady-state without growth. Fig 4B shows the evolution of pore fluid pressure during growth.

Because growth occurs on a rigid cylinder, the radial and axial displacements are zero for all time and also the strain from total deformation is always zero (not pictured). There is a nonzero growth strain from the elastic deformation (not pictured) that causes a buildup of stress. The effective stress is zero during consolidation, because during consolidation both the total and elastic deformations are zero. During material growth, on the other hand, a nonzero elastic deformation forces the material to respect the boundary conditions (here, rigidity). This elastic deformation causes effective stresses to build up in the material.

**Fig 5:**
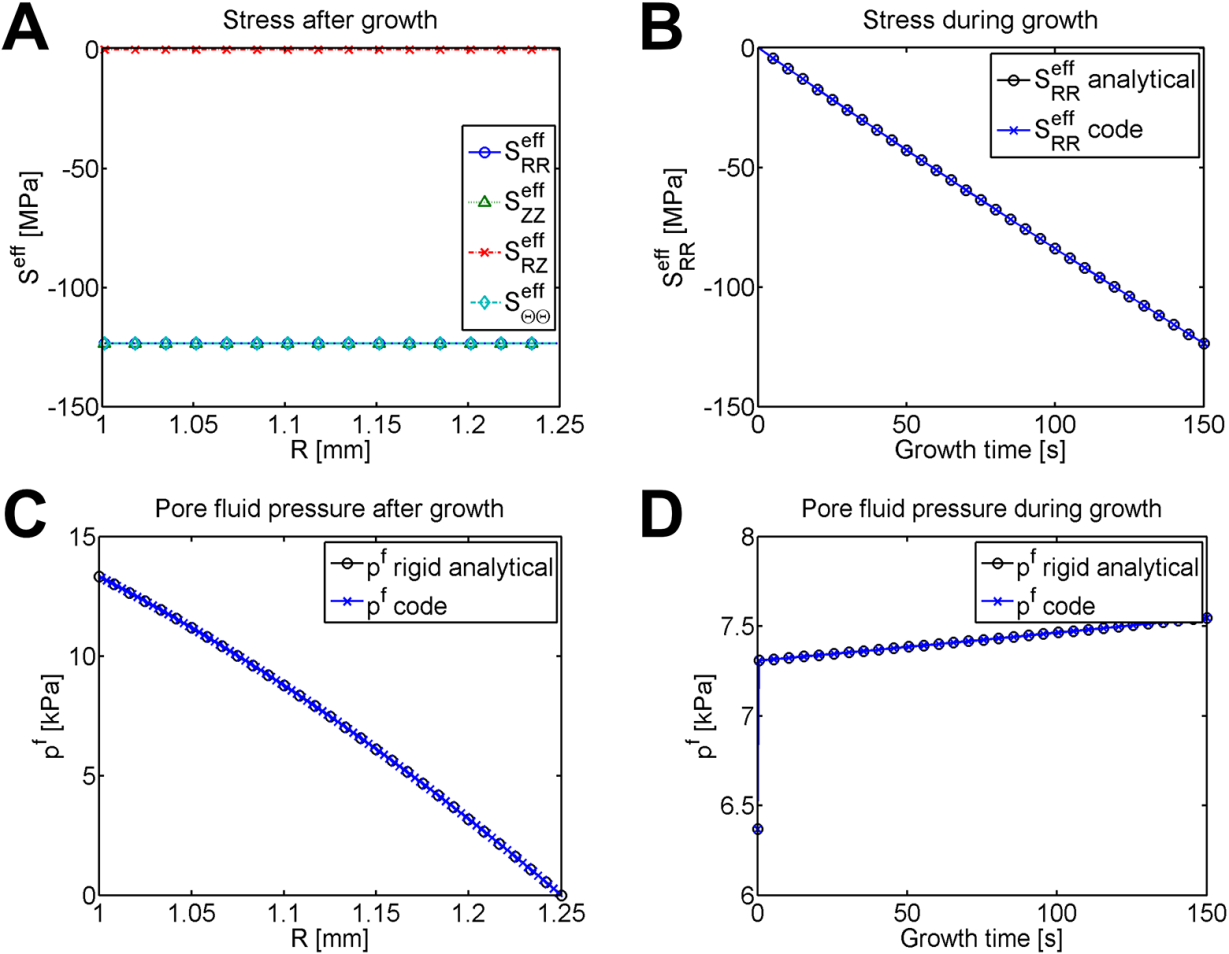
Stress and pore fluid pressure for time-driven solid-only growth of an internally pressurized, rigid cylinder. (A) Effective Second Piola-Kirchhoff stress in a pressurized, rigid cylinder after growth. (B) The effective radial stress from the code matches the analytical solution during growth. All elements have identical stress; radial stress is plotted for an element located at *R* = 1.121 *mm*. Effective axial and hoop stresses appear identical (not shown). (C) Pore fluid pressure matches the analytical solution after growth. Pore fluid pressure (plotted in *kPa*) is non-zero, but four orders of magnitude lower than the material stresses (plotted in *MPa*). (D) Pore fluid pressure matches the analytical solution during growth, and is plotted for an element located at *R* = 1.121 *mm*. The jump when growth begins occurs because *α* = 0 without growth, and when growth begins, the growth rate instantly changes to *α* > 0.

Final stresses are pictured in Fig 5A. The effective stress caused by growth dwarfs the pore fluid pressure, so both the total and effective stress appear very similar (total stress not shown). Because of isotropic growth, the effective stress is the same in all of the primary directions while the shear stress is zero. The development of stress during growth is plotted for radial stress in Fig 5B against the analytical value. Because the stress builds up homogeneously within the material, only a single element is plotted. The negative value of the stress indicates that the material is under compression, as expected from constrained growth.

Fig 5C plots the final pore fluid pressure after growth, which matches well with the analytical solution. The pore fluid pressure during growth is plotted for a single element in Fig 5D and lines up well with the exact analytical solution for pore fluid pressure during growth. The jump at the beginning of Fig 5D occurs because when growth begins, the growth rate instantly changes from *α* = 0 to *α* > 0.

**Fig 6:**
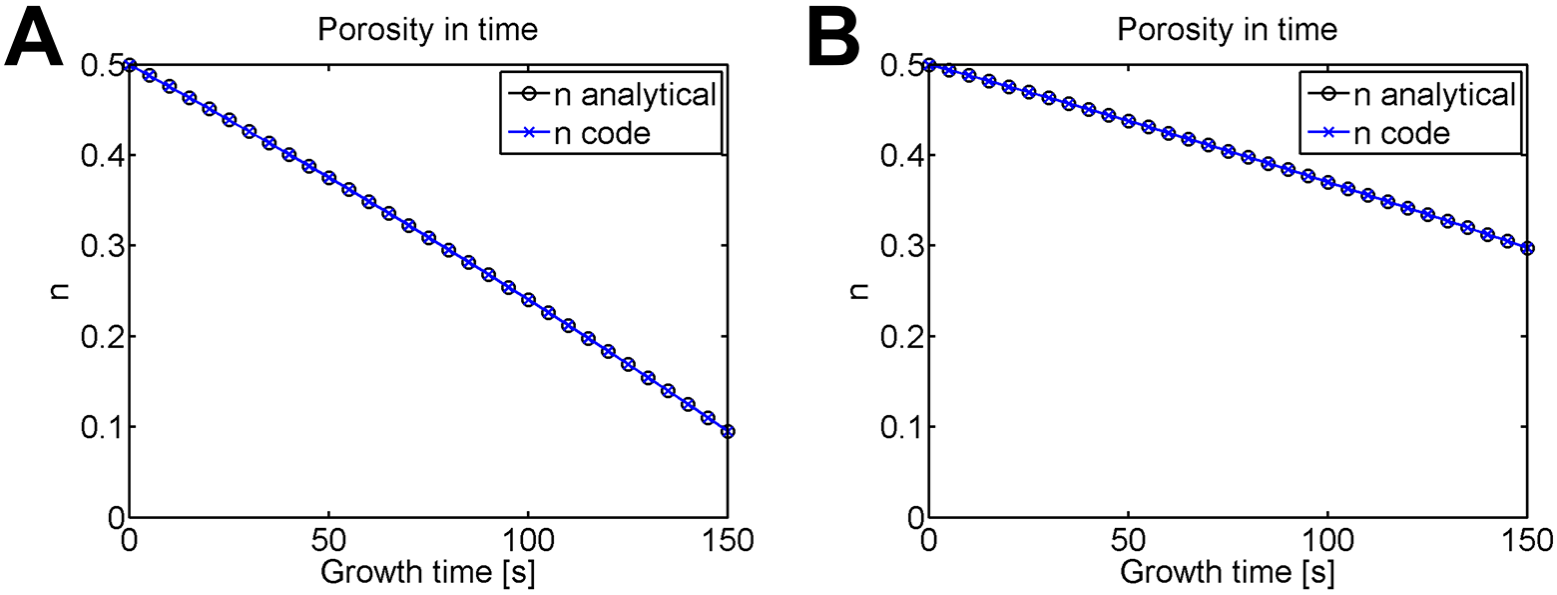
Comparison of porosity for solid-only and solid/fluid mass sources with time-driven growth in an internally pressurized, rigid cylinder. The porosity is plotted during growth time for (A) solid-only growth (${\bar{\rho }}^{s}=1$) and (B) solid/fluid growth (${\bar{\rho }}^{s}=0.5$). In each model, all elements have identical porosity; plotted for an element located at *R* = 1.121 *mm*.

The only value that differs between the solid-only and solid/fluid growth is the porosity, plotted during growth time for both cases in Fig 6. Before growth, the porosity remains constant at 0.5, because there is no deformation. For both cases, the porosity change during growth is nonlinear. Because solid-only growth adds purely solid material, the porosity drops faster than the case with a combination solid/fluid source.

### Stress-dependent growth in pressurized HE vs. MPHETS cylinder

We next simulate stress-dependent growth in a pressurized artery to compare the traditional HE model with our new growing MPHETS model. We apply a nonlinear stress-dependent growth law governed by Eqs (76) and (78). The model adds solid-only material, so ${\bar{\rho }}^{s}=1$; growth parameters are listed in Table 3. The artery is modeled as a strip of 320 elements, with a time step of 50 *s*. The cylinder is pressurized with 5 loading steps, consolidated for a single step, and then 1000 growth steps are applied. In this example, growth takes place simultaneously with fluid and species transport over a period of approximately 14 hours.

**Table 3:** Growth law parameters for stress-dependent growth/resorption of an internally pressurized cylinder.

*τ*^±^	*ϑ*^max^	*ϑ*^min^	*γ*^+^	*γ*^−^	${\bar{\rho }}^{s}$ [unitless]
1e11	1.2	0.8	2	3	1

All parameters are unitless.

**Fig 7:**
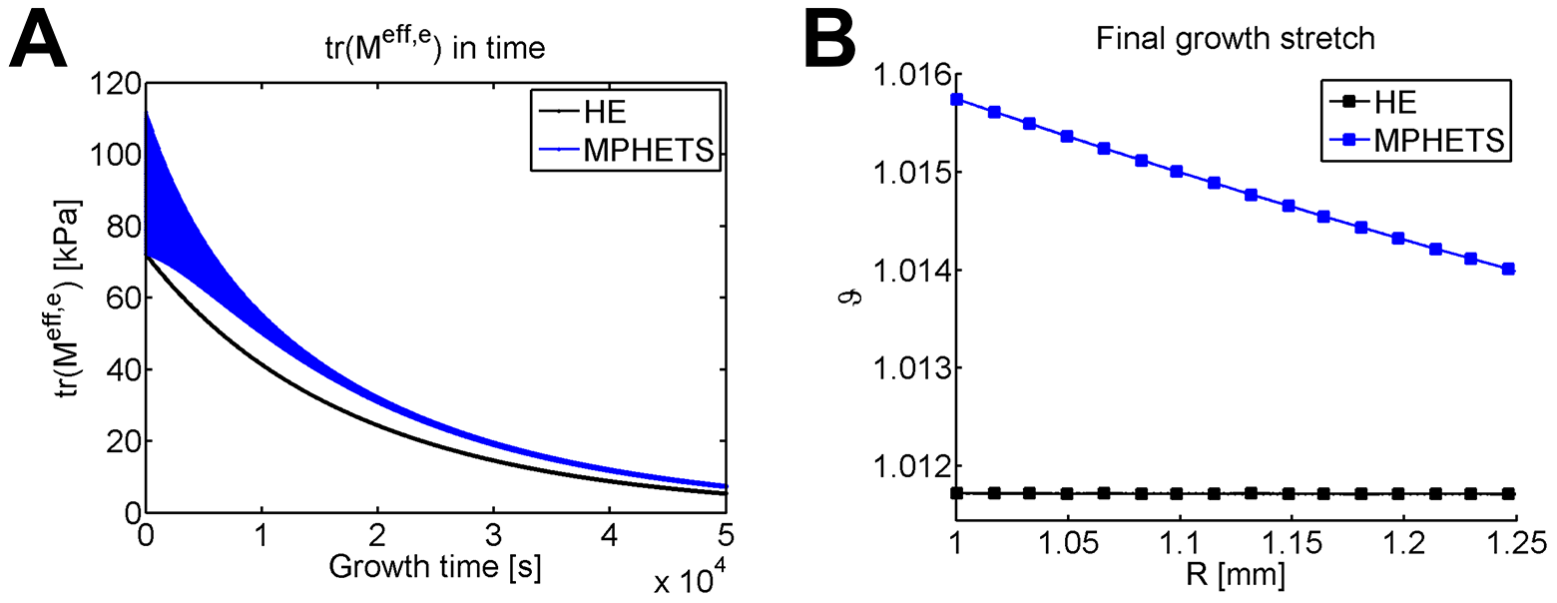
Comparison of stress and growth stretch for HE and MPHETS models of stress-driven growth in an internally pressurized cylinder. (A) Trace of the effective, elastic Mandel stress during growth and (B) final growth stretch in HE and MPHETS models after 1000 growth steps. For both figures, stress is plotted for Gauss points averaged to the center node of adjacent triangles.

Fig 7A shows the Mandel stress plotted for all elements in the model as a function of time. Note that because of the density of the mesh, this appears to be a solid area. Due to the interstitial fluid, the trace of the effective Mandel stress is nonuniform in the MPHETS model. Thus, the MPHETS model stress displays a larger area than the HE model. As growth occurs, the trace of the effective Mandel stress in the MPHETS model becomes more uniform; note that the blue area in Fig 7A decreases with time. Before growth, the trace of the effective Mandel stress in the MPHETS model varies between 72 *kPa* and 112 *kPa*; after 1000 growth steps, it varies between 7.3 *kPa* and 7.5 *kPa*. During growth, the trace of the Mandel stress decreases in both models (shown in Fig 7A) as growth allows the material to adjust in response to residual stresses. Throughout growth, the trace of the Mandel stress is higher in the MPHETS model than in the HE model (shown in Fig 7A), which causes a nonuniform, higher degree of growth in the MPHETS model (plotted in Fig 7B).

**Fig 8:**
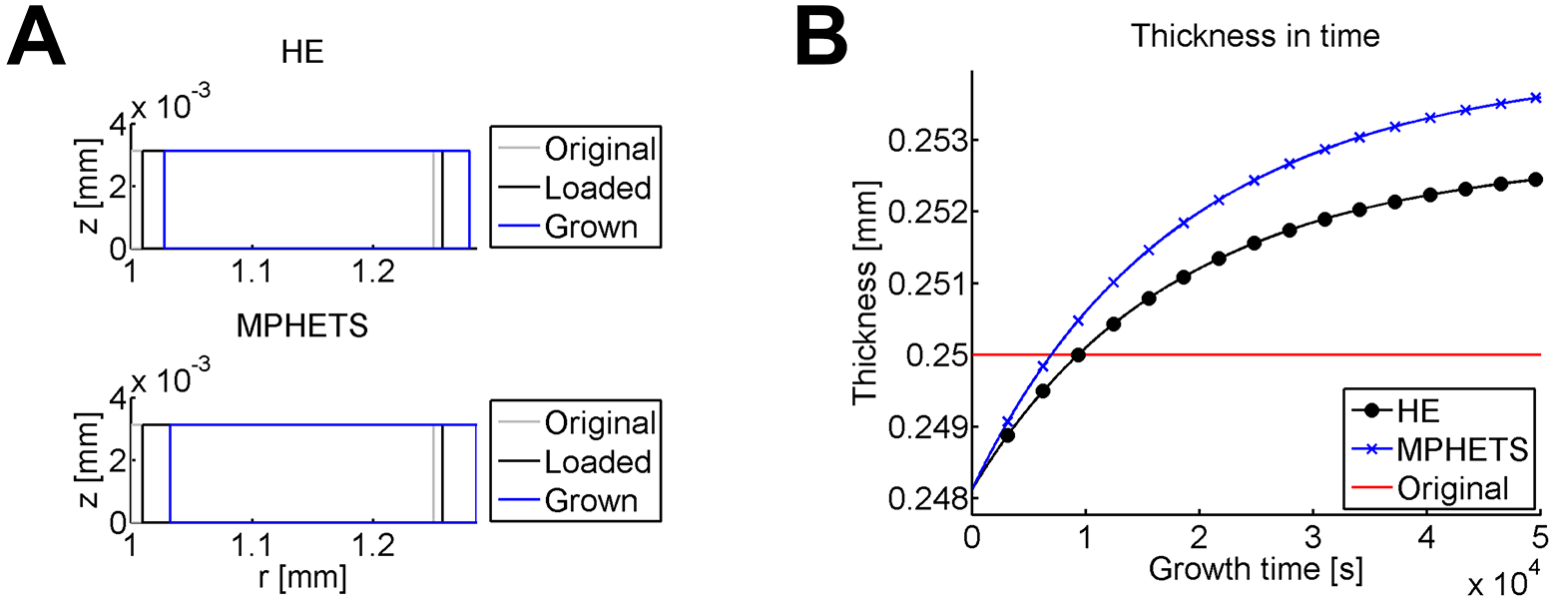
Displacement and thickness for HE and MPHETS models of stress-driven growth in an internally pressurized cylinder. (A) Original, loaded, and final displacements after growth for HE (top) and MPHETS (bottom) models; (B) evolution of thickness in time during 1000 growth steps. For comparison, the initial thickness of the model before loading is also plotted (shown in red).

**Fig 9:**
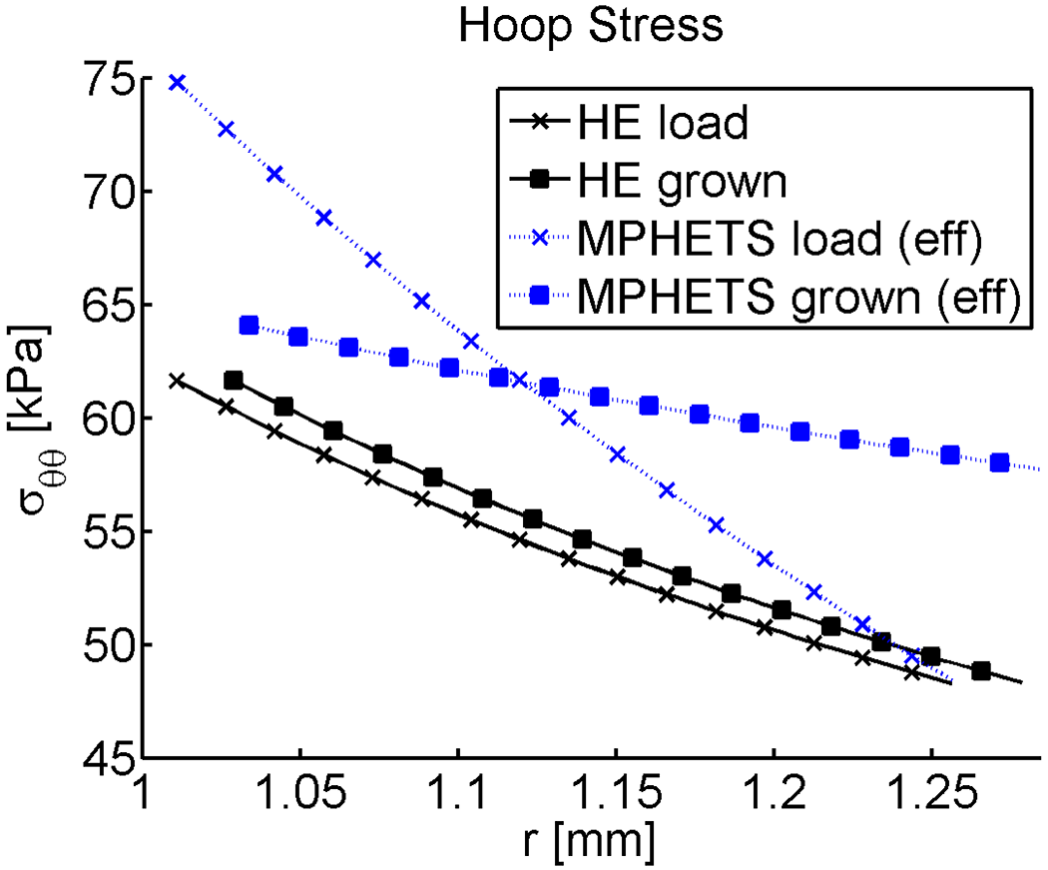
Cauchy hoop stress for loaded and grown states of HE and MPHETS models of stress-driven growth in an internally pressurized cylinder. The plot shows Cauchy stress for the HE model, and effective Cauchy stress for the MPHETS model. Stress is plotted for Gauss points averaged to the center node of adjacent triangles.

The MPHETS and HE models grow differently. The displacement for each mesh and evolution of thickness in time are shown in Fig 8. After loading and consolidation are complete, the displacements are very similar, and the model thickness is likewise very similar. Note that the total thickness has decreased from the original state because loading causes the material to compress. The MPHETS model grows more than the HE model, and hence, becomes a thicker cylinder. In addition, the hoop stresses for each model are different, as seen in Fig 9. For the HE model, the loaded and final states have a similar gradient in hoop stress. In contrast, the MPHETS model has a much steeper gradient in effective hoop stress after loading than the HE model, but after growth occurs, the effective hoop stress becomes more uniform than the hoop stress in the HE model. The buildup of residual stresses due to growth is expected to provide a more uniform stress gradient to resident smooth muscle cells in the media of arterial tissue [52–59].

### Concentration-dependent growth and resorption in an pressurized MPHETS cylinder with a changing ratio of convective to diffusive forces

Lastly, we model concentration-dependent growth in a pressurized artery. To consider the effect of the balance of convective and diffusive forces, we change the diffusivity of the chemical species, which represents a change in drug. The same growth problem is modeled with the experimental value from [43] where *d*^*cc*^ = 4.55*e* − 14 *m*^4^/(*N* ⋅ *s*) (larger Péclet-like number, henceforth called large P) and also with *d*^*cc*^ = 4.55*e* − 12 *m*^4^/(*N* ⋅ *s*) (smaller Péclet-like number, henceforth called small P).

The balance of the ratio of convective forces to diffusive forces creates a Péclet-like number [43, 60] *β* that governs the transient path as well as steady-state distribution of a chemical species, where *β* = (*k*^*ff*^ *b*^*cf*^)/*d*^*cc*^. In this simulation, growth depends explicitly on the chemical concentration. As time progresses, the fluid potential gradient causes fluid flux, which then convects the chemical species across the material. This changing chemical concentration is governed by the Péclet-like number, which thus strongly affects the amount of growth. In addition, as the material grows, additional volume in an element results in a lower chemical concentration which in turn reduces the amount of growth caused by a chemically-driven growth model.

The growth law is given by Eq (75) where *c*_thresh_ is chosen such that it lies in between the minimal and maximal concentrations experienced by the material. For both of these cases, solid-only growth is assumed, so the normalized solid growth density ${\bar{\rho }}^{s}=1$. The parameters *ϑ*^max^ and *ϑ*^min^ bound the growth stretch *ϑ*. In particular, *ϑ*^min^ ensures the porosity is always admissible. Growth parameters are located in Table 4.

**Table 4:** Growth law parameters for concentration-dependent growth/resorption.

*τ*^±^	*c*_thresh_ [*mol*/*m*^3^]	*ϑ*^max^	*ϑ*^min^	*γ*^+^	*γ*^−^	${\bar{\rho }}^{s}$
200	0.009185	1.2	0.8	2	3	1

All parameters are unitless with the exception of the concentration threshold.

For each case, a 160-element mesh is run for five loading steps followed by 2000 growth steps, with time step of *dt* = 50 *s*. Growth takes place simultaneously with fluid and species transport as the model simulates a period of approximately 28 hours.

**Fig 10:**
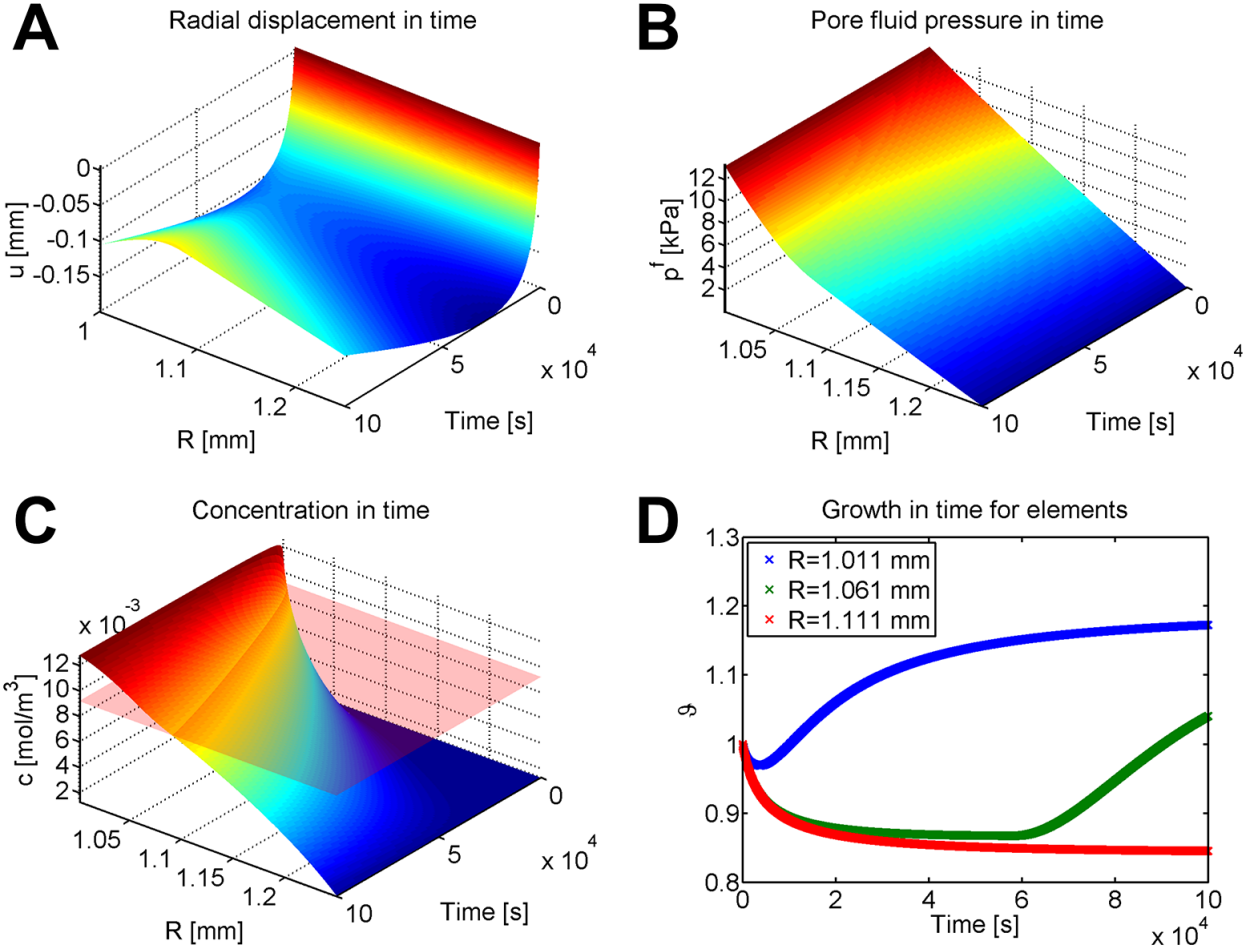
Displacement, pore fluid pressure, concentration, and growth stretch for nonlinear concentration-driven growth in an internally pressurized cylinder. Values plotted for the case of larger Péclet-like number. (A) Displacement in time. Note that displacement initially increases a very small amount due to the pressurization of the vessel, then decreases for all elements due to resorption, followed by an increase due to growth. (B) Pore fluid pressure during growth. (C) Concentration during growth. For comparison, (C) has a transparent red plane that shows the level of *c*_thresh_ for all time. (D) Growth of three specific elements located near the middle of the material are tracked in time. All three elements experience a small amount of resorption at the beginning of growth time, because the concentration is initially below the threshold value. Note that after this initial dip, the innermost element (blue) experiences growth, the outermost element (red) experiences resorption for the entire time, and the middle element (green) experiences resorption followed by growth, ultimately experiencing net growth.

Results for the large P case are plotted in Fig 10. Recall that this case mimics the experimental data for diffusivity from [43]. As depicted in Fig 10A, the displacement at first decreases for all elements, then increases. The pore fluid pressure changes smoothly in time (Fig 10B), decreasing on each individual element due to the changing mesh. Concentration also changes smoothly, as seen in Fig 10C. For both the pore fluid pressure and concentration, plotting on the deformed mesh results in behavior similar to the consolidated state without growth (not pictured). The concentration threshold (plotted as a transparent red surface in Fig 10C) causes a complex growth response, as seen in Fig 10D. As the elements gain or lose material, the concentration across each element changes. Elements near the internal edge of the cylinder always experience growth, while elements at the external edge always experience resorption. However, as the entire material grows and resorbs, the concentration in the center elements changes from lower than *c*_thresh_ (causing resorption) to the higher than *c*_thresh_ (causing growth). Thus, elements near the center of the cylinder will experience material resorption during the first few time steps, followed by growth. This explains the time-dependent radial displacement in Fig 10A; the displacement is the most negative near the beginning of the simulation, while most of the elements are still experiencing resorption.

**Fig 11:**
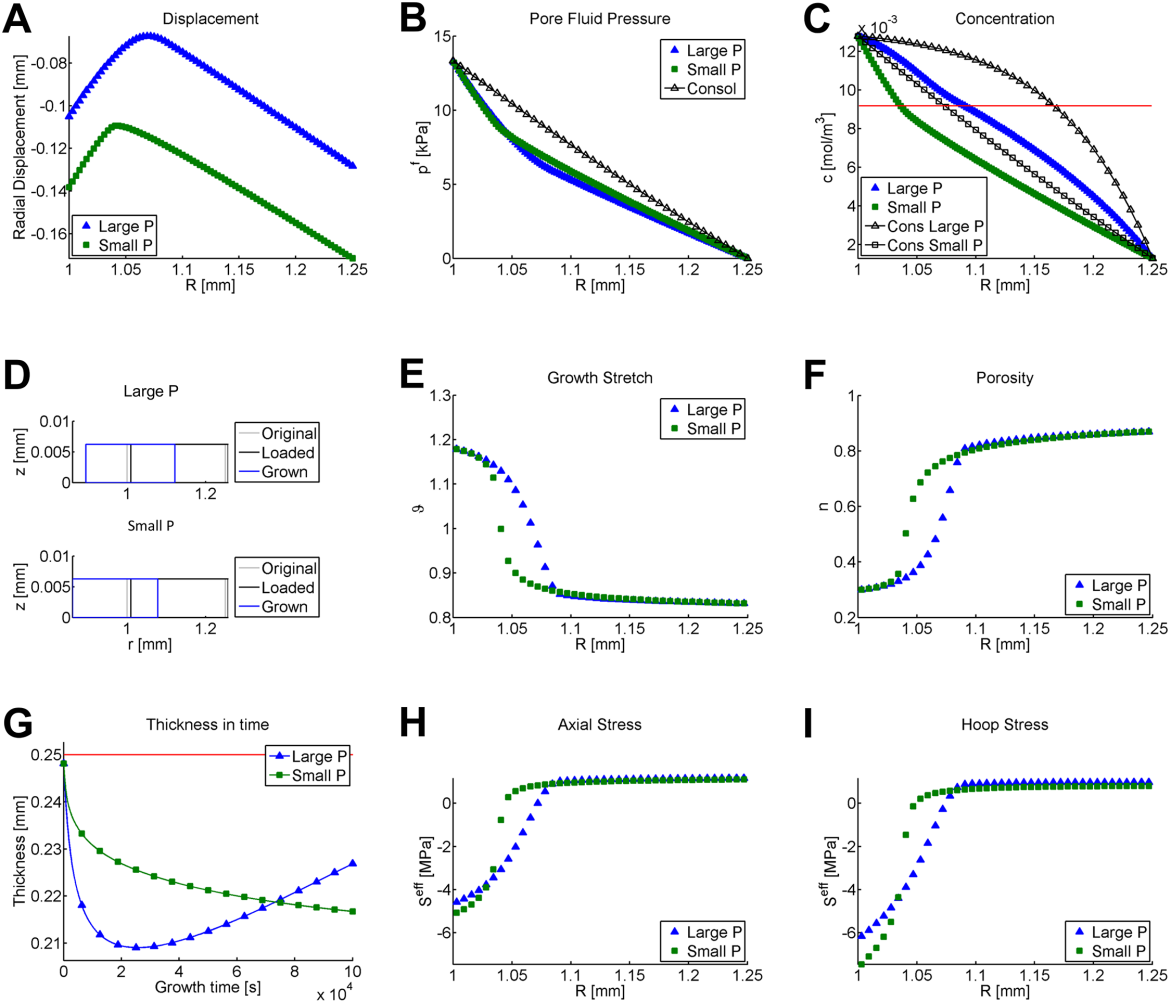
Final displacements, growth stretch, porosity, and stresses for nonlinear concentration-driven growth in an internally pressurized cylinder. Comparison of same growth law for same amount of time, with two different diffusivities. Small P indicates *d*^*cc*^ = 4.55*e* − 12 *m*^2^/*s* while Large P indicates *d*^*cc*^ = 4.55*e* − 14 *m*^2^/*s*. Unless otherwise noted, the final values are shown. (A) Displacement, (B) pore fluid pressure, (C) chemical concentration, (D) original, consolidated, and final displacements after growth for both models, (E) growth stretch, (F) porosity, (G) evolution of thickness in time during 2000 growth steps, (H) effective second Piola-Kirchhoff axial stress, and (I) effective second Piola-Kirchhoff hoop stress. In panel (A), the displacements are plotted at every node. In panels (B) and (C), values are plotted at every Gauss point. For comparison purposes, panel (B) also plots the consolidated pore fluid pressure for the exact solution of a rigid cylinder; this value is the same for both diffusivities. Also for comparison, panel (C) shows the consolidated chemical concentrations for both larger and smaller Péclet-like numbers. Note that a smaller diffusivity corresponds to a higher Péclet-like number and hence, a higher final concentration profile. The red line on panel (C) is the concentration threshold *c*_thresh_. The red line on panel (G) plots the initial thickness of the cylinder prior to loading. Panels (E,F,H,I) are averaged to the center node of adjacent triangles.

Changing the diffusivity *d*^*cc*^ for the small P case alters the distribution and time course of the concentration gradient, which fundamentally changes the distribution of displacement, pore fluid pressure, growth stretch, porosity, strain, and stresses. A comparison of the final outputs is located in Fig 11.

Fig 11A shows the displacements for both cases. The sharp bend in the displacements (and indeed, the discontinuous behavior displayed in many of the panels in Fig 11) is due to the dichotomy of growth and resorption forced by the concentration threshold value. While the displacement after 2000 growth steps is negative for both cases, the displacement for the small P case is more negative than for the large P case. During growth, the small P case experiences continuously decreasing displacement for all elements, unlike the large P case which experiences decreasing followed by increasing displacement. This difference in displacement is caused by the differing concentration distributions shown in Fig 11C. The concentration distribution for the large P case causes more elements to be above the threshold value (shown in red), so more elements experience growth.

The axial and hoop stresses (Fig 11H and 11I) are negative along the inside of the cylinder where the material experiences compression (due to material growth), while the outside of the material experiences tension (due to material resorption). Recall that element movement is constrained in the axial direction. Thus, isotropic growth builds up negative axial stress (compression) while isotropic resorption has the opposite effect.

The growth stretch values and porosity are plotted in Fig 11E and 11F. Fig 11F shows that after 2000 growth steps, the porosity is very different on the left and right sides of the material. The original porosity was 0.5, indicating half fluid and half solid. At the end of the time period, elements on the inside of the cylinder have a porosity lower than the original value (and are mostly solid) while elements on the outside of the cylinder have a porosity higher than the original value (and are mostly fluid). The porosity values correspond strongly with the final values of *ϑ*, in Fig 11E. Parts of the material that experienced a net amount of growth (*ϑ* > 1) have a higher solid content, because growth added solid material. Parts of the material that experienced a net amount of material resorption (*ϑ* < 1) similarly have a lower solid content due to solid resorption. Note that the small P case has more elements nearing the minimal stretch than the large P case (see Fig 11E). This is because the concentration gradient in the small P case causes more elements to be below the threshold than the large P case, and thus more elements experience resorption. This also causes the small P case to have more elements with high porosity (high fluid content).

Thus, changing the ratio of convective to diffusive forces has a large impact on nearly all the results of the growth problem. While both cases result in a thinner cylinder with a smaller external radius compared to the original ungrown cylinder, the large P case results in a thicker cylinder with a larger external radius than the small P case (Fig 11D and 11G). Furthermore, at the end of 2000 growth steps the large P case is trending to a thicker, wider cylinder, while the small P case continues to decrease in both thickness and external radius.

## Discussion

### Summary of approach

A significant contribution of this work is combining the theories of MPHETS and volumetric growth. In this manuscript we built upon the preliminary work of Harper [39, 40] to present a more complete method of modeling soft tissue growth by combining the theories of mixed porohyperelasticity [1, 3, 14] with volumetric growth [6, 16–18]. The MPHETS conservation equations were extended to include the effect of a mass source term. Both solid-only and solid/fluid source terms were considered. In addition to growth modifying the effective stress of the material, this manuscript included the effect of growth on porosity and the fluid and species conservation equations. Multiple growth laws were introduced, including time-, stress-, and concentration-dependent growth. To demonstrate the potential of GMPHETS theory, a finite element framework was introduced and several example problems were discussed using on experimental data for material geometry and parameters [43, 49]. These test problems included comparison with an analytical solution for time-dependent growth in a rigid cylinder. Other more complicated growth problems included stress-dependent growth and concentration-dependent growth.

### Summary of key numerical results

For time-dependent growth of a porohyperelastic rigid cylinder, the added volumetric term shifts the fluid potential to the right. For a rigid problem, adding volume does not cause total deformation; it can only displace the fluid in the material. Because the porosity is the ratio of fluid to total volume, adding solid-only material results in a lower porosity than a combined solid/fluid source term. For a rigid PHE problem with constant permeability, the single difference between adding solid-only material and a combination of solid/fluid material is the change in porosity of the material. Future work should consider permeability that changes based on the porosity, which would provide an additional difference in pore fluid pressure due to growth.

For stress-dependent growth, we have demonstrated that the growth outcome is strongly affected by model type. In a porohyperelastic model, the pore fluid pressure bears mechanical loading, which changes the effective stress experienced by the cells, and thus changes the growth response of the tissue. For growth of a cylinder representing an artery, the MPHETS model experiences a higher amount of growth than the HE model. For both the HE and MPHETS models, growth results in a thicker cylinder which reduces stress gradients. However, the effective hoop stress distribution after growth in an MPHETS model is noticeably more uniform than the hoop stress after growth in an HE model. Thus, the MPHETS model predicts a smaller gradient in hoop stress distribution across the thickness of the model. Other works have shown that grown arteries have a flattened hoop stress distribution [52–59].

For concentration-dependent growth, where chemical concentration may represent a drug or some naturally occurring signaling factor, changing the material parameters has a large effect on growth. For example, it is known that drug distribution is a function of the ratio of convective to diffusive forces [60]. This ratio shifts the evolution and steady-state distribution of a chemical concentration, and thus greatly affects the behavior of chemically-dependent growth. For the problems considered, the combined effects of growth and resorption dynamically change the radius of the cylinder. The change in element size actively shifts the mesh and changes the chemical concentration, which feeds back into the growth loop. Thus, some elements in the model experience a regime of resorption followed by growth. Because of the maximal and minimal stretch values enforced for the model, the cylinder experiences a finite amount of growth. After some large number of time steps, the cylinder experiences two effects: the inside of the cylinder, which experienced growth, is under compression while the outside of the cylinder, which experienced material resorption, is under tension. For a smaller value of diffusivity, the delineation between the ‘inside’ and ‘outside’ shifts to the right, following the concentration distribution. After growth, the cylinder with a higher Péclet-like number (smaller diffusivity) results in a thicker cylinder with a larger external radius than the cylinder with a lower Péclet-like number.

### Limitations and future work

The stress- and concentration-dependent growth laws presented in this work cause either growth or resorption depending on whether the material concentration was above or below a single value. Future work should include changing the growth law to have a larger non-growth tolerance. For example, perhaps material resorption only occurs for concentrations below *c*_threshLow_; no growth occurs for *c*_threshLow_ < *c* < *c*_threshHigh_; and growth occurs for concentrations above *c*_threshHigh_. Another example would be to study the case of growth only, or resorption only; this would model monotonic effects of some drug or chemical signaling factor.

Another limitation of the model introduced in this paper is that all of the constitutive models and growth laws considered are assumed to be isotropic. A more complex model could also include anisotropy and deformation dependent parameters. For example, for illustration purposes we have assumed that the MPHETS parameters (permeability, convection coupling coefficient, and diffusivity) are both constant and isotropic in the Eulerian coordinate system. However, this may not be the case for large strain problems, because the porosity of a material depends on deformation and growth (*n* = *f*(*J*, *n*_*o*_, *ϑ*)) and smaller pores could decrease the permeability and diffusivity of the material. Moreover, an anisotropic model would be capable of handling more complex constitutive behavior. Other works have previously considered directional growth in a solid material, such as Göktepe et al. [6] and Buganza Tepole et al. [9]. These directionally-anisotropic growth models necessitate a more complicated growth pull-back but still have an explicit inverse which simplifies numerical computation [6, 9]. Later work may address the case of strain-dependent porosity, permeability, and diffusivity or consider anisotropic material parameters and/or anisotropic growth. Eventually, a three-dimensional code could possibly be integrated with imaging to produce a patient-specific growth model. In addition, future work should also simultaneously model the combination of stress- and concentration-dependent growth, which are both likely involved in soft tissue growth and remodeling.

While the use of the mechano-chemical potentials as primary variables allows for fluid pressure and concentration jumps at material interfaces, this choice requires the definition of arbitrary baseline values and restricts the values of concentration to be nonzero. Some authors choose to use alternate primary variables that do not carry these restrictions, such as the exponential chemical potential defined in Sun et al. [4]. We feel that an arbitrary choice of the baseline value is not a problem, because the values of the primary variables are merely shifted upwards or downwards with the choice of the baseline concentration. However, if one wishes to simulate arbitrarily small concentrations, one may prefer alternate potentials such as those used in [4].

An additional limitation of this work is that only the response of growth has been considered; future work should also incorporate remodeling, which involves changing constitutive properties. For example, growth could change the density or stiffness of the material; or change the orientation of collagen fibers (which would change the directionality of the stress response); or increase the fiber density. To change the assumption of density preservation during growth would require careful consideration of the effect on conservation of mass. Himpel et al. [17] considered remodeling for a solid-only material via a change in density; this would be a good starting point for adding remodeling to the theory. Changing the material properties or orientation of collagen fibers would likely require a more complicated mechanism for tracking constituents including mass turnover and survival fractions as in constrained mixture theory [61–64]. The addition of evolving reference configurations [61] or a microstructurally-based growth law [65] would also incorporate the effect of stretch on each constituent. These laws could model even more complicated growth and remodeling in a porohyperelastic biological system with chemical transport.

In summary, the GMPHETS theory developed in this manuscript is a good starting point for more complex growth models. Rather than modeling soft tissue as a solid material, it is important to consider the effect of an interstitial fluid. Because an interstitial fluid bears stress, the distinction between hyperelastic and porohyperelastic models becomes especially important for stress-dependent growth. Future applications can provide more realistic and complex simulation of how soft tissues grow in response to a complex combination of time, stress, and chemical concentration. This work will hopefully provide a point of departure for scientists to better understand the evolution of soft tissue diseases.

## Supporting Information

S1 Appendix A brief summary of MPHETS theory. (PDF)

S2 Appendix Derivation of conservation equations for GMPHETS. (PDF)

S3 Appendix Weak form of the conservation equations for GMPHETS. (PDF)

S4 Appendix Derivation of incremental form of conservation equations and assembly of global tangent modulus. (PDF)

S5 Appendix Derivation of stresses and pore fluid pressure for time-dependent growth in a pressurized rigid cylinder. (PDF)

S6 Appendix Table of densities used in GMPHETS. (PDF)
