# Supplementary material for: A Finite Element Model for Mixed Porohyperelasticity with Transport, Swelling, and Growth
Source: PLoS One. 2016 Apr 14;11(4):e0152806. doi: 10.1371/journal.pone.0152806 (PMC4831841; doi:10.1371/journal.pone.0152806)
Supplement: S1 Appendix — (PDF) [file pone.0152806.s001.pdf]

S1 Appendix

Theory and material constitutive laws for mixed porohyperelasticity with transport and swelling (MPHETS)

In this section, we present a brief background on traditional MPHETS theory (without growth). For more detail, we refer the reader to [1, 3, 14, 40, 41].

Kinematics The material displacement of a point from X_i to x_i is given as $u_i = x_i - X_i$ where x_i is the Eulerian coordinate of the material and X_i is the Lagrangian coordinate of the material. Then the deformation gradient F_{ij} may be defined as $F_{ij} = \frac{\partial x_i}{\partial X_j}$ with Jacobian $J = \det(F_{ij})$. The spatial rate of deformation of a point is given by $D_{ij} = \frac{1}{2}\left(\frac{\partial v_i}{\partial x_j} + \frac{\partial v_j}{\partial x_i}\right)$ for material velocity $v_i = \frac{du_i}{dt}$ [1].

The material is considered to be a combination of solid, fluid, and species, but the volume of the species can be neglected compared to that of the solid and fluid. Then the differential volume element is given by $dV = dV^s + dV^f$ for dV^s the differential volume of the solid and dV^f the differential volume of the fluid.

Conservation Equations in Eulerian Coordinates Linear momentum must be conserved. Without body forces, the quasi-static conservation of linear momentum may be written as $\frac{\partial \sigma_{ij}}{\partial x_i} = 0$, for σ_{ij} the Cauchy stress tensor. The conservation of angular momentum results in the symmetry of the Cauchy stress tensor: $\sigma_{ij} = \sigma_{ji}$.

Combining the conservation of mass of both solid and fluid with the incompressibility condition yields a relationship between the fluid velocity gradient and the time-dependent deformation of the material. In Eulerian coordinates, $D_{ii} + D^{fr}_{kk} = \frac{\partial v_i}{\partial x_i} + \frac{\partial j^{fr}_k}{\partial x_k} = 0$ where D_{ii} is the dilatational velocity strain of the material and D^{fr}_{kk} refers to the relative fluid velocity strain. Henceforth, this equation and all of its derivatives will be referred to as the fluid conservation equation. The relative fluid flux is given in terms of the porosity n, the velocity of the fluid v^f_i, and the velocity of the solid v^s_i (which is equal to the velocity of the material v_i). The relative fluid flux is defined as $j^{fr}_i = n(v^f_i - v^s_i)$, where porosity n is defined as $n = \frac{dV^f}{dV}$. The fluid conservation equation converts between the divergence of relative fluid flux and the divergence of solid velocity. Thus, this condition mandates that dilatational effects of the material are converted directly into fluid strain.

Similarly, the conservation of mass of the species yields a time-dependent expression relating the relative species flux j^{cr} to deformation. In Eulerian coordinates $\frac{\partial j^{cr}_i}{\partial x_i} + \frac{\partial (ncv^s_i)}{\partial x_i} + \frac{\partial (nc)}{\partial t} = 0$ for relative species flux $j^{cr}_i = nc(v^c_i - v^s_i)$ and concentration c.

Using the incompressibility of the solid and fluid constituents, the current porosity may be written as $n = 1 - J^{-1}(1 - n_0)$ for the initial porosity $n_0 = \frac{dV^f_0}{dV_0}$.

Conservation Equations in Lagrangian Coordinates The conservation of linear momentum, fluid conservation equation, and conservation of mass of the species may be

written in Lagrangian coordinates as

$$\frac{\partial T_{ij}}{\partial X_i} = 0, \tag{S.1}$$

$$\frac{\partial \tilde{j}_k^{fr}}{\partial X_k} + JH_{ij}\dot{E}_{ij} = 0, \tag{S.2}$$

$$\frac{\partial \tilde{j}_k^{cr}}{\partial X_k} + JH_{ij}\dot{E}_{ij}c + Jn\dot{c} = 0, \tag{S.3}$$

where $T_{ij} = F_{ip}S_{pj}$ is the first Piola-Kirchhoff stress tensor; by the correspondence rules the Lagrangian relative fluid and species fluxes are defined as $\tilde{j}_k^{fr} = JF_{ki}^{-1}j_i^{fr}$ and $\tilde{j}_k^{cr} = JF_{ki}^{-1}j_i^{cr}$ [13, 15]; and by definition Finger's strain tensor $H_{ij} = F_{ip}^{-1}F_{jp}^{-1}$ and Green strain $E_{ij} = \frac{1}{2}\left(F_{pi}F_{pj} - \delta_{ij}\right)$ for δ_{ij} the identity tensor [1]. Note that here, a tilde denotes a Lagrangian quantity.

Constitutive Relationships The total stress is comprised of an effective and fluid part, and may be expressed in Lagrangian coordinates as

$$T_{ij} = F_{ip}S_{pj} = F_{ip}(S_{pj}^{\text{eff}} - JH_{pj}p^f), \tag{S.4}$$

where F_{ip} is the deformation gradient, S_{pj} is the second Piola-Kirchhoff stress, S_{pj}^{eff} is the effective second Piola-Kirchhoff stress, $H_{pj} = F_{pk}^{-1}F_{jk}^{-1}$ is Finger's strain tensor and p^f is the pore fluid pressure. The effective stress is the stress in the material after subtracting out the stress on the fluid constituent.

This effective stress is assumed to have a hyperelastic form, such that the stress can be derived from an energy function W^{eff}, where

$$S_{ij}^{\text{eff}} = \frac{\partial W^{\text{eff}}}{\partial E_{ij}}. \tag{S.5}$$

The fluid-species interactions may be described by the Onsager equations, which are a generalized version Darcy's and Fick's laws and couple the species and the pore fluid pressure. The Onsager equations are

$$\tilde{j}_i^{fr} = -\tilde{L}_{ij}^{ff}\frac{\partial \tilde{\mu}^{f^*}}{\partial X_j} - \tilde{L}_{ij}^{fc}\frac{\partial \tilde{\mu}^{c^*}}{\partial X_j}, \tag{S.6}$$

$$\tilde{j}_i^{cr} = -\tilde{L}_{ij}^{cf}\frac{\partial \tilde{\mu}^{f^*}}{\partial X_j} - \tilde{L}_{ij}^{cc}\frac{\partial \tilde{\mu}^{c^*}}{\partial X_j}, \tag{S.7}$$

where $\tilde{L}_{ij}^{ff}, \tilde{L}_{ij}^{fc}, \tilde{L}_{ij}^{cf}, \tilde{L}_{ij}^{cc}$ are given material parameters that can be calculated from the MPHETS parameters of porosity k^{ff}, convection coupling coefficient $b^{fc} = b^{cf}$, and diffusivity d^{cc} (assumed to be isotropic in the Eulerian frame) [1, 40]. The Lagrangian $\tilde{L}_{ij}$ parameters may be written as

$$\tilde{L}_{ij}^{ff} = JH_{ij}k^{ff}, \tag{S.8}$$

$$\tilde{L}_{ij}^{fc} = JH_{ij}\left(k^{ff}b^{fc}c\right) = JH_{ij}\left(cb^{cf}k^{ff}\right) = \tilde{L}_{ij}^{cf}, \tag{S.9}$$

$$\tilde{L}_{ij}^{cc} = JH_{ij}\left(\frac{c}{\bar{R}\theta}d^{cc} + cb^{cf}k^{ff}b^{fc}c\right), \tag{S.10}$$

where $\bar{R} = 8.31$ $(J/K/mol)$ is the universal gas constant and θ is the material temperature (assumed to be constant) [1, 40].

Secondary solution variables The primary and secondary variables are linked by the standard mechano-chemical potentials [3, 40]. The fluid potential $\tilde{\mu}^{f^*}$ is defined as

$$\tilde{\mu}^{f^*} = p^f + p_0^o - \bar{R}\theta\phi^c c, \tag{S.11}$$

for p_0^o a baseline osmotic potential and ϕ^c the osmotic coefficient of the species in the material, and the chemical potential $\tilde{\mu}^{c^*}$ is defined as

$$\tilde{\mu}^{c^*} = \mu_o^c + \bar{R}\theta \log(\gamma_{\mathrm{mat}}^c c), \tag{S.12}$$

where μ_o^c is a baseline chemical potential and γ^c is the activity coefficient of the species in the material.
