# Supplementary material for: A Finite Element Model for Mixed Porohyperelasticity with Transport, Swelling, and Growth
Source: PLoS One. 2016 Apr 14;11(4):e0152806. doi: 10.1371/journal.pone.0152806 (PMC4831841; doi:10.1371/journal.pone.0152806)
Supplement: S2 Appendix — (PDF) [file pone.0152806.s002.pdf]

S2 Appendix

Derivation of conservation equations for GMPHETS

The conservation and constitutive equations for MPHETS with growth (GMPHETS) differ from the traditional MPHETS equations because of the added volume from the mass source term as well as stress modified by growth. In this section the Lagrangian conservation equations with growth are derived from the base of traditional MPHETS theory (the traditional theory is briefly explained in S1 Appendix). For more details on the derivation, the reader is referred to Armstrong [41].

The conservation of linear momentum remains the same as the non-growth case: in Lagrangian coordinates,

$$\frac{\partial T_{ij}}{\partial X_i} = 0. \tag{S.13}$$

The fluid residual is derived from the Lagrangian conservation of mass for the incompressible solid and fluid. For growth, the conservation of mass of the solid and the conservation of mass of the fluid now have source terms which enter the second conservation equation. Summing the Eulerian open system conservation of mass for a solid and open system conservation of mass for a fluid, using the assumption of incompressibility, and transforming to Lagrangian coordinates, the second conservation equation becomes

$$\frac{\partial \tilde{j}_k^{fr}}{\partial X_k} + JH_{ij}\dot{E}_{ij} - \frac{\mathcal{R}_0^s}{\rho_T^s} - \frac{\mathcal{R}_0^f}{\rho_T^f} = 0. \tag{S.14}$$

with Lagrangian solid mass source term $\mathcal{R}_0^s$, fluid mass source term $\mathcal{R}_0^f$, and ρ_T^α the true density of constituent α in kg/m^3. The true density is defined as $\rho_T^\alpha = dM^\alpha/dV_0^\alpha$ for dM^α the initial mass and dV_0^α the initial volume of constituent α. The units of both mass sources are $kg/(m^3 \cdot s)$. Thus, source term $\mathcal{R}_0^\alpha/\rho_T^\alpha$ has units of $1/s$. The source terms are completely determined by the (given) time evolution of growth.

Recall from the manuscript that the mass source rate for isotropic growth are given by $3\rho_0^{s^*}\vartheta^2\dot{\vartheta}$ and $3\rho_0^{f^*}\vartheta^2\dot{\vartheta}$ where $\rho_0^{s^*}, \rho_0^{f^*}$ are the solid and fluid densities preserved during growth (see equations (22), (32), and (35)). Then the mass source term $\mathcal{R}_0^\alpha/\rho_T^\alpha$ does not depend on the mass of the solid. For either solid-only growth that preserves the true density of the solid or solid/fluid growth that preserves the initial apparent densities, the sum of density specific source terms is given by $3\vartheta^2\dot{\vartheta}$ (equations (24) and (40)). Then the fluid conservation from equation (S.14) may be written simply as

$$\frac{\partial \tilde{j}_k^{fr}}{\partial X_k} + JH_{ij}\dot{E}_{ij} - 3\vartheta^2\dot{\vartheta} = 0. \tag{S.15}$$

Comparing this to the original conservation equation (S.2), one may see that growth has added a volumetric term to the fluid conservation equation.

Because growth changes the material porosity, the conservation of mass for the species differs from the non-growth case. Thus, we re-derive the conservation equation for growth. We begin with the conservation of mass of the species without source terms. The density of the species is given by $\rho^c = nc$, which gives the mass of species per unit volume of the fluid. Then the Lagrangian conservation of mass of the species may be written as

$$\frac{D}{Dt}(Jnc) + \frac{\partial \tilde{j}_k^{cr}}{\partial X_k} = 0. \tag{S.16}$$

This equation may then be split further using the chain rule to yield

$$\frac{\partial \tilde{j}_k^{cr}}{\partial X_k} + \dot{\widehat{Jn}}c + Jn\dot{c} = 0. \tag{S.17}$$

In general, for constant density growth the porosity is given by equation (44), repeated here.

$$n = 1 - J^{-1}(1 - n_0) + J^{-1}\left[\frac{\rho_0^{s^*}}{\rho_T^s}(\vartheta^3 - 1)\right]. \tag{S.18}$$

To simplify notation, we define the (constant, scalar) normalized solid growth density as $\bar{\rho}^s = \rho_0^{s^*}/\rho_T^s$. Rearranging this equation and taking the total time derivative results in the relationship

$$\dot{\widehat{Jn}} = \dot{J} - 3\bar{\rho}^s\vartheta^2\dot{\vartheta}. \tag{S.19}$$

The time derivative of J is given by $\dot{J} = JH_{ij}\dot{E}_{ij}$ [14]. Then using equation (S.19), equation (S.17) may be written as the conservation of mass of the species in Lagrangian terms as

$$\frac{\partial \tilde{j}_k^{cr}}{\partial X_k} + JH_{ij}\dot{E}_{ij}c - 3\bar{\rho}^s\vartheta^2\dot{\vartheta}c + Jn\dot{c} = 0, \tag{S.20}$$

where $\bar{\rho}^s = \rho_0^{s^*}/\rho_T^s$ is the normalized solid growth density. Comparing this to the original conservation of species mass in equation (S.3), one may see that a growth term has been added and furthermore, that this growth term depends on the growth density. Thus, the species conservation equation will behave differently for different growth laws.

The effective stress is the only constitutive equation modified by growth, and is found in the main body of the paper. The other constitutive equations (63)-(69) remain the same as the non-growth case.
