# Supplementary material for: A Finite Element Model for Mixed Porohyperelasticity with Transport, Swelling, and Growth
Source: PLoS One. 2016 Apr 14;11(4):e0152806. doi: 10.1371/journal.pone.0152806 (PMC4831841; doi:10.1371/journal.pone.0152806)
Supplement: S3 Appendix — (PDF) [file pone.0152806.s003.pdf]

S3 Appendix

Weak form of conservation equations

The finite element method solves for the variables in the weak sense. Integrating equation (54) against the test function $\mathbf{N}_u$ results in the weak conservation of linear momentum, given by

$$\mathbf{\Psi}_u = \int \widehat{\mathbf{B}}_u^T \mathbf{S} dV_o - \mathbf{P}_u^{\text{ext}} \stackrel{\text{d}}{=} 0, \tag{S.21}$$

where $\widehat{\mathbf{B}}_u^T$ is the traditional nonlinear strain matrix in axisymmetry such that $\Delta\mathbf{E} = \widehat{\mathbf{B}}_u^T \Delta\mathbf{u}$ for material displacements $\mathbf{u}$ (e.g., see Ch. 5 of Zienkiewicz and Taylor's monograph [45]). The traction boundary condition is implemented through $\mathbf{P}_u^{\text{ext}} = \int \mathbf{N}_u^T \mathbf{T} \widehat{\mathbf{n}}_0 dA_0$ for $\mathbf{T} = \mathbf{FS}$, unit normal vector $\widehat{\mathbf{n}}_0$, and differential original area dA_0 [14].

Next consider the fluid equation. For simplicity of notation, define the total mass source as

$$\overline{\mathcal{R}}_0 = \frac{\mathcal{R}_0^s}{\rho_T^s} + \frac{\mathcal{R}_0^f}{\rho_T^f}. \tag{S.22}$$

Integrating the fluid conservation equation (55) over the original volume against the test function $\mathbf{N}_f$ and then using divergence theorem and Onsager equations, the weak form of the fluid conservation equation may be written as

$$\begin{aligned} \Psi_f = &- \int \mathbf{B}_f^T \left(\tilde{\mathbf{L}}^{ff} \mathbf{B}_f \overline{\mu}^{f^*} + \tilde{\mathbf{L}}^{fc} \mathbf{B}_c \overline{\mu}^{c^*} \right) dV_0 \\ &- \int \mathbf{N}_f^T J \mathbf{H}^T \widehat{\mathbf{B}}_u dV_0 \dot{\bar{\mathbf{u}}} - \mathbf{P}_f^{\text{ext}} + \int \mathbf{N}_f^T \left(\overline{\mathcal{R}}_0 \right) dV_0 = 0, \end{aligned} \tag{S.23}$$

where $\mathbf{B}_f$ and $\mathbf{B}_c$ are linear strain operators (from the gradient in test functions $\mathbf{N}_f, \mathbf{N}_c$), and $\mathbf{P}_f^{\text{ext}} = \int \mathbf{N}_f^T \tilde{\mathbf{j}}^{fr} \widehat{\mathbf{n}}_0 dA_0$ is the fluid flux at the boundary. The bar above the primary variables $\bar{\mathbf{u}}, \overline{\mu}^{f^*}, \overline{\mu}^{c^*}$ indicates that the variables are evaluated at the nodes. For certain parameters, it is possible that growth may occur so slowly that the source term can be neglected. For completeness, we leave it as part of the full formulation. Note that the source term is analogous to a body forcing term in a solid finite element problem.

Similarly, integrating the conservation of species mass from equation (56) against the test function $\mathbf{N}_c$, applying the divergence theorem and using the definition of the solid strain rate, the weak form of the species conservation of mass may be written as

$$\begin{aligned} \Psi_c = &- \int \mathbf{B}_c^T \left(\tilde{\mathbf{L}}^{cf} \mathbf{B}_f \overline{\mu}^{f^*} + \tilde{\mathbf{L}}^{cc} \mathbf{B}_c \overline{\mu}^{c^*} \right) dV_0 - \mathbf{P}_c^{\text{ext}} - \int \mathbf{N}_c^T \left(J \mathbf{H}^T \widehat{\mathbf{B}}_u \dot{\bar{\mathbf{u}}} c^g \right) dV_0 \\ &+ \int \mathbf{N}_c^T \left(3 \bar{\rho}^s \vartheta^2 \dot{\vartheta} c^g \right) dV_0 - \int \mathbf{N}_c^T \left(J n \dot{c}^g \right) dV_0 = 0, \end{aligned} \tag{S.24}$$

for $\mathbf{B}_c$ the traditional linear strain operator and surface species flux defined as $\mathbf{P}_c^{\text{ext}} = \int \mathbf{N}_c^T \tilde{\mathbf{j}}^{cr} \widehat{\mathbf{n}}_0 dA_0$. The bar above the primary variables $\bar{\mathbf{u}}, \overline{\mu}^{f^*}, \overline{\mu}^{c^*}$ indicates that the variables are evaluated at the nodes, while the superscript g on the concentration indicates that it is evaluated at the Gauss point.
