# Supplementary material for: A Finite Element Model for Mixed Porohyperelasticity with Transport, Swelling, and Growth
Source: PLoS One. 2016 Apr 14;11(4):e0152806. doi: 10.1371/journal.pone.0152806 (PMC4831841; doi:10.1371/journal.pone.0152806)
Supplement: S4 Appendix — (PDF) [file pone.0152806.s004.pdf]

S4 Appendix

This appendix contains the derivation of the incremental form of conservation equations and assembly of the global tangent modulus. This is followed by the derivations for the incremental form of the growth source terms.

Incremental residual for global Newton-Raphson iterations

In this section we develop the incremental residual for global Newton-Raphson iterations.

Increment in conservation of linear momentum Applying the effective stress principle, the increment of the residual from equation (S.21) is given by

$$\Delta\mathbf{\Psi}_u = \int \Delta\widehat{\mathbf{B}}_u^T\ \mathbf{S}dV_o + \int \widehat{\mathbf{B}}_u^T\ \Delta\mathbf{S}dV_o - \Delta\mathbf{P}_u^{\text{ext}} \stackrel{\text{d}}{=} 0. \tag{S.25}$$

By the effective stress principle, this may also be written as

$$\Delta\mathbf{\Psi}_u = \int \Delta\widehat{\mathbf{B}}_u^T\mathbf{S}dV_o + \int \widehat{\mathbf{B}}_u^T \left(\Delta\mathbf{S}^{\text{eff}} - \Delta(J\mathbf{H}p^f)\right) dV_o - \Delta\mathbf{P}_u^{\text{ext}}. \tag{S.26}$$

Because stress depends on both the growth and porohyperelastic response of the material, the increment in the effective stress now contains two terms: an elastic component, in which growth is held fixed, and a growth component, in which the total deformation is held fixed. Recall that the increment represents the total derivative, and yields the tangent modulus.

The tangent modulus will be very similar to [6, 17], but modified for GMPHETS. Then

$$\begin{aligned}\Delta\mathbf{S}^{\text{eff}} &= \left(\left.\frac{\partial\mathbf{S}^{\text{eff}}}{\partial\mathbf{E}}\right|_{\mathbf{F}^g} + \frac{\partial\mathbf{S}^{\text{eff}}}{\partial\vartheta}\otimes\left.\frac{\partial\vartheta}{\partial\mathbf{E}}\right|_{\mathbf{F}}\right)\Delta\mathbf{E}\\ &= \left(\underbrace{\left.\frac{\partial\mathbf{S}^{\text{eff}}}{\partial\mathbf{E}}\right|_{\mathbf{F}^g}}_{(1)} + \underbrace{\frac{\partial\mathbf{S}^{\text{eff}}}{\partial\mathbf{F}^g} : \frac{\partial\mathbf{F}^g}{\partial\vartheta}\otimes\left.\frac{\partial\vartheta}{\partial\mathbf{E}}\right|_{\mathbf{F}}}_{(2)}\right)\Delta\mathbf{E}, = (\mathbb{L}^{eg})\,\Delta\mathbf{E}\end{aligned} \tag{S.27}$$

where the chain rule has been applied [6]. Recall that stress depends on total deformation; and also, if the material deforms through growth, the stress changes. The first term is sometimes called the elastic tangent modulus and provides the porohyperelastic contribution to the tangent modulus while the second term is sometimes referred to as the growth tangent modulus and provides the growth contribution. Note that the effect of growth is still included in the first term, and likewise, elastic deformation is included in the second term.

Consider the first term of equation (S.27). For this term of the chain rule, the growth deformation $\mathbf{F}^g$ is fixed. Then for isotropic growth, we have

$$\left.\frac{\partial\mathbf{S}^{\text{eff}}}{\partial\mathbf{E}}\right|_{\mathbf{F}^g} = \left.\frac{\partial}{\partial\mathbf{E}}\left(\frac{1}{\vartheta^2}\mathbf{S}^{\text{eff},e}\right)\right|_{\mathbf{F}^g} = \frac{1}{\vartheta^2}\left(2\frac{\partial\mathbf{S}^{\text{eff},e}}{\partial\mathbf{C}^e}\right) : \left.\frac{\partial\mathbf{C}^e}{\partial\mathbf{C}}\right|_{\mathbf{F}^g} = \frac{1}{\vartheta^4}\mathbb{L}^e \tag{S.28}$$

where we have used the fact that $\mathbf{C}^e = \mathbf{F}^{g-T}\mathbf{C}\mathbf{F}^{g-1} = \mathbf{C}/\vartheta^2$ [6]. The fourth order tensor $\mathbb{L}^e$ is the tangent modulus of the elastic deformation for the particular form of strain energy density chosen, e.g. it takes the form of equation (72).

The second term of equation (S.27) will only appear if $\dfrac{\partial\vartheta}{\partial\mathbf{E}}$ is nonzero. For example, if growth depends only on time or concentration, there is not an explicit dependence of growth on strain and the entire second term is zero. In contrast, for growth dependent on the stress, strain, or deformation gradient, this term has a nonzero value.

Then the tangent is written for isotropic time- or concentration-dependent growth as

$$\mathbb{L}^{eg} = \left.\frac{\partial\mathbf{S}^{\text{eff}}}{\partial\mathbf{E}}\right|_{\mathbf{F}^g} = \frac{1}{\vartheta^4}\mathbb{L}^e \tag{S.29}$$

which is a symmetric tensor due to the symmetry of $\mathbb{L}^e$.

For isotropic stress-dependent growth, we summarize the theory from Göktepe et al. [6] modified for the effective stress of GMPHETS. The second term of equation (S.27) requires the tangent modulus for local Newton iteration, detailed in the Numerical algorithm section of this paper. The local tangent modulus for stress-dependent growth of an arbitrary growth function (given in equation (4) as $\dot{\vartheta} = k_\vartheta(\vartheta)\phi^g(t, c, \mathbf{F}, \mathbf{M}^{\text{eff}}, \ldots)$) is defined in [6] as

$$K_\vartheta = \left[1 - \left(k_\vartheta\frac{\partial\phi^g}{\partial\vartheta} + \frac{\partial k_\vartheta}{\partial\vartheta}\phi^g\right)\Delta t\right]. \tag{S.30}$$

K_ϑ is scalar valued, so it appropriate to write $1/K_\vartheta$ for the inverse. Then with a slight rewrite of the derivative in the last term of equation (S.27) via $\partial(\cdot)/\partial\mathbf{E} = 2\partial(\cdot)/\partial\mathbf{C}$, the tangent for isotropic stress-dependent growth may be written as

$$\begin{aligned}\mathbb{L}^{eg} &= \left.\frac{\partial\mathbf{S}^{\text{eff}}}{\partial\mathbf{E}}\right|_{\mathbf{F}^g} + 2\left.\frac{\partial\mathbf{S}^{\text{eff}}}{\partial\mathbf{F}^g} : \frac{\partial\mathbf{F}^g}{\partial\vartheta}\otimes\frac{\partial\vartheta}{\partial\mathbf{C}}\right|_{\mathbf{F}} \\ &= \frac{1}{\vartheta^4}\mathbb{L}^e - \frac{4}{\vartheta^5}\frac{k_\vartheta\Delta t}{K_\vartheta}\left(\mathbf{S}^{\text{eff},e} + \frac{1}{2}\mathbb{L}^e : \mathbf{C}^e\right)\otimes\left(\frac{1}{2}\mathbf{C}^e : \mathbb{L}^e + \mathbf{S}^{\text{eff},e}\right),\end{aligned} \tag{S.31}$$

which is very similar to the equation found in [6]. Additionally, while in general growth constitutive moduli need not be symmetric, Göktepe et al. note that equation (S.31) (written in terms of the elastic second Piola-Kirchhoff stress) is symmetric [6]; this is still symmetric for GMPHETS using the effective, elastic second Piola-Kirchhoff stress.

For GMPHETS theory, the tangent also includes a term from the pore fluid pressure. Taking the increment in the $J\mathbf{H}$ term results in the tensor term

$$\Delta(J\mathbf{H}) = J\left(\mathbf{H}\otimes\mathbf{H} - \mathbf{H}\,\overline{\otimes}\,\mathbf{H} - \mathbf{H}\,\underline{\otimes}\,\mathbf{H}\right), \tag{S.32}$$

for tensor products $\mathbf{A}\otimes\mathbf{B} = A_{ij}B_{kl}$; $\mathbf{A}\,\overline{\otimes}\,\mathbf{B} = A_{ik}B_{jl}$; and $\mathbf{A}\,\underline{\otimes}\,\mathbf{B} = A_{il}B_{jk}$. Note that Finger's strain tensor $\mathbf{H}$ is equal to $\mathbf{C}^{-1}$, so the tensor expression here takes on a similar form to the tensor used in $\mathbb{L}^e$ (equation (72)).

Finally, the increment in the solid residual (for isotropic growth not depending on

strain) is very similar to the solid residual without growth:

$$\begin{aligned}\Delta\mathbf{\Psi}_u &= \int \widehat{\mathbf{B}}_u^T \left(\mathbb{L}^{eg} - Jp^f \left(\mathbf{H}\otimes\mathbf{H} - \mathbf{H}\,\overline{\otimes}\,\mathbf{H} - \mathbf{H}\,\underline{\otimes}\,\mathbf{H}\right)\right)\widehat{\mathbf{B}}_u dV_o \Delta\bar{\mathbf{u}} \\ &+ \int \Delta\widehat{\mathbf{B}}_u^T \mathbf{S} dV_o - \int \widehat{\mathbf{B}}_u^T J\mathbf{H} dV_o \Delta p^f - \Delta\mathbf{P}_u^{\text{ext}},\end{aligned} \tag{S.33}$$

where the only difference from traditional MPHETS theory is the modification of the elastic tangent modulus to $\mathbb{L}^{eg}$.

In matrix form, equation (S.33) may be written as

$$\Delta\mathbf{\Psi}_u = [\mathbf{k}_{uu}^{\text{effs}}]\Delta\bar{\mathbf{u}} + [\mathbf{k}_{uu}^{(S)}]\Delta\bar{\mathbf{u}} + [\mathbf{k}'_{uf}]\Delta p^{fg} - \Delta\mathbf{P}_u^{\text{ext}} = 0, \tag{S.34}$$

for matrices

$$\begin{aligned}[\mathbf{k}_{uu}^{\text{effs}}] &= \int \widehat{\mathbf{B}}_u^T \left(\mathbb{L}^{eg} - Jp^f \left(\mathbf{H}\otimes\mathbf{H} - \mathbf{H}\,\overline{\otimes}\,\mathbf{H} - \mathbf{H}\,\underline{\otimes}\,\mathbf{H}\right)\right)\widehat{\mathbf{B}}_u dV_o, \\ [\mathbf{k}_{uu}^{(S)}] &= \int \Delta\widehat{\mathbf{B}}_u^T \mathbf{S} dV_o, \qquad [\mathbf{k}'_{uf}] = \int \Delta\widehat{\mathbf{B}}_u^T \mathbf{S} dV_o - \int \widehat{\mathbf{B}}_u^T J\mathbf{H} dV_o.\end{aligned} \tag{S.35}$$

where $[\mathbf{k}_{uu}^{(S)}]$ is the initial stress matrix coming from the increment in operator $\widehat{\mathbf{B}}_u$ [46, 47]. Combining terms, this may also be written as

$$\Delta\mathbf{\Psi}_u = [\mathbf{k}_{uu}]\Delta\bar{\mathbf{u}} + [\mathbf{k}'_{uf}]\Delta p^{fg} - \Delta\mathbf{P}_u^{\text{ext}} = 0, \qquad \text{for } [\mathbf{k}_{uu}] = [\mathbf{k}_{uu}^{(S)}] + [\mathbf{k}_{uu}^{\text{effs}}]. \tag{S.36}$$

Increment in fluid conservation equation We choose to neglect $\Delta\tilde{\mathbf{L}}^{\alpha\beta}$ terms when we linearize. These incremental $\Delta\tilde{\mathbf{L}}^{\alpha\beta}$ terms are therefore not included in the tangent modulus, but any effects from the increment will appear in the residual calculation. Rearranging, the increment of equation (S.23) may be written as

$$\begin{aligned}\Delta\Psi_f = &\left[-\int \mathbf{N}_f^T J\mathbf{H}^T \widehat{\mathbf{B}}_u dV_0\right]\Delta\dot{\mathbf{u}} \\ &+ \left[-\int \mathbf{N}_f^T J\dot{\mathbf{u}}^T \widehat{\mathbf{B}}_u^T (\mathbf{H}\otimes\mathbf{H} - \mathbf{H}\overline{\otimes}\mathbf{H} - \mathbf{H}\underline{\otimes}\mathbf{H})\widehat{\mathbf{B}}_u dV_0\right]\Delta\bar{\mathbf{u}} \\ &+ \left[-\int \mathbf{N}_f^T J\dot{\mathbf{u}}^T \Delta\widehat{\mathbf{B}}_u^T \mathbf{H} dV_0\right]\Delta\bar{\mathbf{u}} + \left[-\frac{\partial\mathbf{P}_f^{\text{ext}}}{\partial\bar{\mathbf{u}}}\right]\Delta\bar{\mathbf{u}} \\ &+ \left[-\int \mathbf{B}_f^T \tilde{L}^{ff}\mathbf{B}_f dV_0\right]\Delta\bar{\mu}^{f^*} + \left[-\int \mathbf{B}_f^T \tilde{L}^{fc}\mathbf{B}_c dV_0\right]\Delta\bar{\mu}^{c^*} \\ &- \left[\frac{\partial\mathbf{P}_f^{\text{ext}}}{\partial t}\Delta t\right] + \Delta\mathbf{P}^s = 0.\end{aligned} \tag{S.37}$$

In matrix form, this is just

$$\begin{aligned}\Delta\Psi_f = &\left[\mathbf{c}_{fu}\right]\Delta\dot{\mathbf{u}} + \left[\mathbf{k}_{fu}^{JH}\right]\Delta\bar{\mathbf{u}} + \left[\mathbf{k}_{fu}^{MH}\right]\Delta\bar{\mathbf{u}} + \left[\mathbf{k}_{fu}^{Pf}\right]\Delta\bar{\mathbf{u}} \\ &+ \left[\mathbf{k}_{ff}\right]\Delta\bar{\mu}^{f^*} + \left[\mathbf{k}_{fc}\right]\Delta\bar{\mu}^{c^*} - \Delta\widehat{\mathbf{P}}_f^{\text{ext}} + \Delta\mathbf{P}^s = 0,\end{aligned} \tag{S.38}$$

for matrices defined as

$$
\begin{aligned}
[\mathbf{c}_{fu}] &= -\int \mathbf{N}_f^T J \mathbf{H}^T \widehat{\mathbf{B}}_u dV_0, \\
\left[\mathbf{k}_{fu}^{JH}\right] &= -\int \mathbf{N}_f^T J \dot{\mathbf{u}}^T \widehat{\mathbf{B}}_u^T (\mathbf{H}\otimes\mathbf{H} - \mathbf{H}\overline{\otimes}\mathbf{H} - \mathbf{H}\underline{\otimes}\mathbf{H}) \widehat{\mathbf{B}}_u dV_0, \\
\left[\mathbf{k}_{fu}^{MH}\right] &= -\int \mathbf{N}_f^T J \dot{\mathbf{u}}^T \Delta \widehat{\mathbf{B}}_u^T \mathbf{H} dV_0, \qquad \left[\mathbf{k}_{fu}^{Pf}\right] = -\frac{\partial \mathbf{P}_f^{\mathrm{ext}}}{\partial \bar{\mathbf{u}}}, \\
[\mathbf{k}_{ff}] &= -\int \mathbf{B}_f^T \tilde{L}^{ff} \mathbf{B}_f dV_0, \qquad [\mathbf{k}_{fc}] = -\int \mathbf{B}_f^T \tilde{L}^{fc} \mathbf{B}_c dV_0,
\end{aligned} \tag{S.39}
$$

where $\left[\mathbf{k}_{fu}^{MH}\right]$ has a formulation very similar to the initial stress matrix from above. Combining terms, equation (S.38) may be written as

$$
\Delta\Psi_f = [\mathbf{c}_{fu}]\,\Delta\dot{\bar{\mathbf{u}}} + [\mathbf{k}_{fu}]\,\Delta\bar{\mathbf{u}} + [\mathbf{k}_{ff}]\,\Delta\bar{\mu}^{f^*} + [\mathbf{k}_{fc}]\,\Delta\bar{\mu}^{c^*} - \Delta\widehat{\mathbf{P}}_f^{\mathrm{ext}} + \Delta\mathbf{P}^s = 0, \tag{S.40}
$$

where

$$
[\mathbf{k}_{fu}] = \left[\mathbf{k}_{fu}^{JH}\right] + \left[\mathbf{k}_{fu}^{MH}\right] + \left[\mathbf{k}_{fu}^{Pf}\right]. \tag{S.41}
$$

The increment $\Delta\mathbf{P}^s$ will differ based on growth law type. Derivations of the particular forms of $\Delta\mathbf{P}^s$ appear later in this appendix.

Increment in conservation of mass of the chemical species Rearranging equation (S.24) and using the substitution for Jn from equation (S.18), this is just

$$
\begin{aligned}
\Psi_c = &\left[-\int \mathbf{N}_c^T c^g J \mathbf{H}^T \widehat{\mathbf{B}}_u dV_0\right] \dot{\bar{\mathbf{u}}} + \left[-\int \mathbf{N}_c^T \left\{J - (1-n_0) - \bar{\rho}^s(\vartheta^3 - 1)\right\} dV_0\right] \dot{c}^g \\
&+ \left[-\int \mathbf{B}_c^T \tilde{\mathbf{L}}^{cf} \mathbf{B}_f dV_0\right] \bar{\mu}^{f^*} + \left[-\int \mathbf{B}_c^T \tilde{\mathbf{L}}^{cc} \mathbf{B}_c dV_0\right] \bar{\mu}^{c^*} \\
&+ \left[\int \mathbf{N}_c^T \left(3\bar{\rho}^s \vartheta^2 \dot{\vartheta}\right) dV_0\right] c^g - \mathbf{P}_c^{\mathrm{ext}} = 0,
\end{aligned} \tag{S.42}
$$

or equation (S.42) may be rewritten in matrix form as

$$
\Psi_c = [\mathbf{c}_{cu}]\,\dot{\bar{\mathbf{u}}} + \left[\mathbf{c}_{cg}'\right] \dot{c}^g + [\mathbf{k}_{cf}]\,\bar{\mu}^{f^*} + [\mathbf{k}_{cc}]\,\bar{\mu}^{c^*} + \left[\mathbf{k}_{cg,\mathrm{grow}}'\right] c^g - \mathbf{P}_c^{\mathrm{ext}} = 0, \tag{S.43}
$$

for matrices defined as

$$
\begin{aligned}
\left[\mathbf{c}_{cg}'\right] &= -\int \mathbf{N}_c^T \left\{J - (1-n_0) - \bar{\rho}^s(\vartheta^3 - 1)\right\} dV_0, \\
[\mathbf{c}_{cu}] &= -\int \mathbf{N}_c^T c^g J \mathbf{H}^T \widehat{\mathbf{B}}_u dV_0, \qquad [\mathbf{k}_{cf}] = -\int \mathbf{B}_c^T \tilde{\mathbf{L}}^{cf} \mathbf{B}_f dV_0, \\
[\mathbf{k}_{cc}] &= \mathbf{B}_c^T \tilde{\mathbf{L}}^{cc} \mathbf{B}_c dV_0, \qquad \left[\mathbf{k}_{cg,\mathrm{grow}}'\right] = \int \mathbf{N}_c^T \left(3\bar{\rho}^s \vartheta^2 \dot{\vartheta}\right) dV_0.
\end{aligned} \tag{S.44}
$$

The matrix $\left[\mathbf{k}_{cg,\mathrm{grow}}'\right]$ is caused by growth; this matrix is not present in standard MPHETS theory.

The increment of the growth contribution produces two terms:

$$\Delta\left(\left[\mathbf{k}'_{cg,\text{grow}}\right]c^g\right) = \underbrace{\left[\int \mathbf{N}_c^T \Delta\left(3\bar{\rho}^s\vartheta^2\dot{\vartheta}\right)dV_0\right]}_{(1)} c^g + \underbrace{\left[\int \mathbf{N}_c^T\left(3\bar{\rho}^s\vartheta^2\dot{\vartheta}\right)dV_0\right]}_{(2)}\Delta c^g \tag{S.45}$$

The expression in the first term depends on the type of growth considered. Specific forms will be considered in later in this appendix.

So, taking the increment of equation (S.42) and neglecting both the $\Delta\tilde{\mathbf{L}}^{\alpha\beta}$ terms and the follower loading effect on the external forcing, the species residual only contains the following terms:

$$\begin{aligned}\Delta\Psi_c = &\left[-\int \mathbf{N}_c^T c^g J\mathbf{H}^T\widehat{\mathbf{B}}_u dV_0\right]\Delta\dot{\bar{\mathbf{u}}}\\
&+\left[-\int \mathbf{N}_c^T\left\{J-(1-n_0)-\bar{\rho}^s(\vartheta^3-1)\right\}dV_0\right]\Delta\dot{c}^g\\
&+\left[-\int \mathbf{N}_c^T J\mathbf{H}^T\widehat{\mathbf{B}}_u\dot{\bar{\mathbf{u}}}dV_0\right]\Delta c^g+\left[\int \mathbf{N}_c^T\left(3\bar{\rho}^s\vartheta^2\dot{\vartheta}\right)dV_0\right]\Delta c^g\\
&+\left[-\int \mathbf{N}_c^T\left\{c^g\dot{\bar{\mathbf{u}}}^T\widehat{\mathbf{B}}_u^T J\left(\mathbf{H}\otimes\mathbf{H}-\mathbf{H}\,\overline{\otimes}\,\mathbf{H}-\mathbf{H}\,\underline{\otimes}\,\mathbf{H}\right)\widehat{\mathbf{B}}_u\right\}dV_0\right]\Delta\bar{\mathbf{u}}\\
&+\left[-\int \mathbf{N}_c^T c^g J\left(\dot{\bar{\mathbf{u}}}^T\Delta\widehat{\mathbf{B}}_u^T\mathbf{H}\right)dV_0\right]\Delta\bar{\mathbf{u}}+\left[-\int \mathbf{N}_c^T\dot{c}^g J\mathbf{H}^T\widehat{\mathbf{B}}_u dV_0\right]\Delta\bar{\mathbf{u}}\\
&+\left[-\frac{\partial\mathbf{P}_c^{\text{ext}}}{\partial\bar{\mathbf{u}}}\right]\Delta\bar{\mathbf{u}}+\left[-\int \mathbf{B}_c^T\tilde{\mathbf{L}}^{cf}\mathbf{B}_f dV_0\right]\Delta\bar{\mu}^{f^*}\\
&+\left[-\int \mathbf{B}_c^T\tilde{\mathbf{L}}^{cc}\mathbf{B}_c dV_0\right]\Delta\bar{\mu}^{c^*}-\left[\frac{\partial\mathbf{P}_c^{\text{ext}}}{\partial t}\Delta t\right]=0.\end{aligned}\tag{S.46}$$

Growth makes two modifications in the chemical species tangent: in the second term, the substitution for Jn from equation (S.18) includes a growth term, and there is an additional incremental contribution from the growth from equation (S.45) (not shown in the above equation, because it is neglected in the tangent modulus for our model).

In matrix form, equation (S.46) may be written as

$$\begin{aligned}\Delta\Psi_c = &\left[\mathbf{c}_{cu}\right]\Delta\dot{\bar{\mathbf{u}}}+\left[\mathbf{c}'_{cg}\right]\Delta\dot{c}^g+\left[\mathbf{k}'_{cg}\right]\Delta c^g+\left[\mathbf{k}'_{cg,\text{grow}}\right]\Delta c^g+\left[\mathbf{k}_{cu}^{JH}\right]\Delta\bar{\mathbf{u}}\\
&+\left[\mathbf{k}_{cu}^{MH}\right]\Delta\bar{\mathbf{u}}+\left[\mathbf{k}_{cu}^{Jn}\right]\Delta\bar{\mathbf{u}}+\left[\mathbf{k}_{cu}^{Pc}\right]\Delta\bar{\mathbf{u}}+\left[\mathbf{k}_{cf}\right]\Delta\bar{\mu}^{f^*}\\
&+\left[\mathbf{k}_{cc}\right]\Delta\bar{\mu}^{c^*}-\Delta\widehat{\mathbf{P}}_c^{\text{ext}}=0,\end{aligned}\tag{S.47}$$

with matrices from equation (S.44), and with additional matrices defined as

$$\begin{aligned}\left[\mathbf{c}_{cu}\right]=-\int \mathbf{N}_c^T c^g J\mathbf{H}^T\widehat{\mathbf{B}}_u dV_0,\qquad \left[\mathbf{k}'_{cg}\right]=-\int \mathbf{N}_c^T\left\{J-(1-n_0)-\bar{\rho}^s(\vartheta^3-1)\right\}dV_0,\\
\left[\mathbf{k}_{cu}^{JH}\right]=-\int \mathbf{N}_c^T\left\{c^g\dot{\bar{\mathbf{u}}}^T\widehat{\mathbf{B}}_u^T J\left(\mathbf{H}\otimes\mathbf{H}-\mathbf{H}\,\overline{\otimes}\,\mathbf{H}-\mathbf{H}\,\underline{\otimes}\,\mathbf{H}\right)\widehat{\mathbf{B}}_u\right\}dV_0,\\
\left[\mathbf{k}_{cu}^{MH}\right]=-\int \mathbf{N}_c^T c^g J\left(\dot{\bar{\mathbf{u}}}^T\Delta\widehat{\mathbf{B}}_u^T\mathbf{H}\right)dV_0,\\
\left[\mathbf{k}_{cu}^{Jn}\right]=-\int \mathbf{N}_c^T\dot{c}^g J\mathbf{H}^T\widehat{\mathbf{B}}_u dV_0,\qquad \left[\mathbf{k}_{cu}^{Pc}\right]=-\frac{\partial\mathbf{P}_c^{\text{ext}}}{\partial\bar{\mathbf{u}}},\end{aligned}\tag{S.48}$$

where $\left[\mathbf{k}_{cu}^{MH}\right]$ has a formulation very similar to the initial stress matrix from above. Combining like terms and rearranging, this equation may be written as

$$\begin{aligned}\Delta\Psi_c = &\left[\mathbf{c}_{cu}\right]\Delta\dot{\bar{\mathbf{u}}} + \left[\mathbf{c}'_{cg}\right]\Delta\dot{c}^g + \left[\mathbf{k}_{cu}\right]\Delta\bar{\mathbf{u}} + \left[\mathbf{k}_{cf}\right]\Delta\bar{\mu}^{f^*} \\ &+ \left[\mathbf{k}_{cc}\right]\Delta\bar{\mu}^{c^*} + \left[\mathbf{k}'_{cg}\right]\Delta c^g - \Delta\widehat{\mathbf{P}}_c^{\text{ext}} = 0,\end{aligned} \quad \text{(S.49)}$$

for $\left[\mathbf{k}_{cu}\right] = \left[\mathbf{k}_{cu}^{JH}\right] + \left[\mathbf{k}_{cu}^{MH}\right] + \left[\mathbf{k}_{cu}^{Jn}\right] + \left[\mathbf{k}_{cu}^{Pc}\right]$ and $\left[\tilde{\mathbf{k}}'_{cg}\right] = \left[\mathbf{k}'_{cg}\right] + \left[\mathbf{k}'_{cg,\text{grow}}\right]$.

Assemble matrices for primary incremental residuals on an element

Assembly of the tangent modulus is completely analogous to the non-growth case.

The primary incremental residuals may be assembled to form the complete primary residual on an element. By stacking these as vectors and combining matrices, one may write the primary residual on an element as

$$\begin{aligned}\Delta\Psi_e = \begin{bmatrix}\Delta\Psi_u\\ \Delta\Psi_f\\ \Delta\Psi_c\end{bmatrix} = &\begin{bmatrix}0&0&0\\ \mathbf{c}_{fu}&0&0\\ \mathbf{c}_{cu}&0&0\end{bmatrix}\begin{bmatrix}\Delta\dot{\bar{\mathbf{u}}}\\ \Delta\dot{\bar{\mu}}^{f^*}\\ \Delta\dot{\bar{\mu}}^{c^*}\end{bmatrix} + \begin{bmatrix}0&0&0\\0&0&0\\0&0&\mathbf{c}'_{cg}\end{bmatrix}\begin{bmatrix}\Delta\dot{\mu}^{ce}\\ \Delta\dot{p}^f\\ \Delta\dot{c}^g\end{bmatrix}\\ &+ \begin{bmatrix}\mathbf{k}_{uu}&0&0\\ \mathbf{k}_{fu}&\mathbf{k}_{ff}&\mathbf{k}_{fc}\\ \mathbf{k}_{cu}&\mathbf{k}_{cf}&\mathbf{k}_{cc}\end{bmatrix}\begin{bmatrix}\Delta\bar{\mathbf{u}}\\ \Delta\bar{\mu}^{f^*}\\ \Delta\bar{\mu}^{c^*}\end{bmatrix}\\ &+ \begin{bmatrix}0&\mathbf{k}'_{uf}&0\\0&0&\mathbf{k}_{fcg}^{G'}\\0&0&\mathbf{k}'_{cg}\end{bmatrix}\begin{bmatrix}\Delta\mu^{ce}\\ \Delta p^f\\ \Delta c^g\end{bmatrix} - \begin{bmatrix}\Delta\widehat{\mathbf{P}}_u^{\text{ext}}\\ \Delta\widehat{\mathbf{P}}_f^{\text{ext}}\\ \Delta\widehat{\mathbf{P}}_c^{\text{ext}}\end{bmatrix} = \begin{bmatrix}0\\0\\0\end{bmatrix}.\end{aligned} \quad \text{(S.50)}$$

Note that the entry $\mathbf{k}_{fcg}^{G'}$ only appears for concentration dependent growth. This contribution will be derived later in this appendix.

Equation (S.50) may also be written in matrix form as

$$\Delta\Psi = \left[\mathbf{c}_{pp}\right]\Delta\dot{\mathbf{p}} + \left[\mathbf{c}_{pg}\right]\Delta\dot{\mathbf{p}}^g + \left[\mathbf{k}_{pp}\right]\Delta\mathbf{p} + \left[\mathbf{k}_{pg}\right]\Delta\mathbf{p}^g - \Delta\widehat{\mathbf{P}}^{\text{ext}} = \mathbf{0}, \quad \text{(S.51)}$$

with vectors

$$\Delta\dot{\mathbf{p}} = \begin{bmatrix}\Delta\dot{\bar{\mathbf{u}}}\\ \Delta\dot{\bar{\mu}}^{f^*}\\ \Delta\dot{\bar{\mu}}^{c^*}\end{bmatrix}, \quad \Delta\dot{\mathbf{p}}^g = \begin{bmatrix}\Delta\dot{\mu}^{ce}\\ \Delta\dot{p}^f\\ \Delta\dot{c}^g\end{bmatrix}, \quad \Delta\mathbf{p} = \begin{bmatrix}\Delta\bar{\mathbf{u}}\\ \Delta\bar{\mu}^{f^*}\\ \Delta\bar{\mu}^{c^*}\end{bmatrix}, \quad \Delta\mathbf{p}^g = \begin{bmatrix}\Delta\mu^{ce}\\ \Delta p^f\\ \Delta c^g\end{bmatrix}, \quad \text{(S.52)}$$

and matrices

$$\begin{aligned}\left[\mathbf{c}_{pp}\right] &= \begin{bmatrix}0&0&0\\ \mathbf{c}_{fu}&0&0\\ \mathbf{c}_{cu}&0&0\end{bmatrix}, & \left[\mathbf{c}_{pg}\right] &= \begin{bmatrix}0&0&0\\0&0&0\\0&0&\mathbf{c}'_{cg}\end{bmatrix},\\ \left[\mathbf{k}_{pp}\right] &= \begin{bmatrix}\mathbf{k}_{uu}&0&0\\ \mathbf{k}_{fu}&\mathbf{k}_{ff}&\mathbf{k}_{fc}\\ \mathbf{k}_{cu}&\mathbf{k}_{cf}&\mathbf{k}_{cc}\end{bmatrix}, & \left[\mathbf{k}_{pg}\right] &= \begin{bmatrix}0&\mathbf{k}'_{uf}&0\\0&0&\mathbf{k}_{fcg}^{G'}\\0&0&\mathbf{k}'_{cg}\end{bmatrix}.\end{aligned} \quad \text{(S.53)}$$

Note that the matrices $\left[\mathbf{c}_{pg}\right]$ and $\left[\mathbf{k}_{pg}\right]$ are not square. This is because they are multiplied by secondary quantities defined at the Gauss points. The secondary residuals will be used to solve for these quantities in terms of the primary variables. The

secondary residuals are assembled in a similar fashion; for details see Armstrong [41].

Assemble matrices for global incremental residuals The elemental tangent matrices are then assembled into the global tangent following a standard finite element assembly procedure.

Incremental form of the mass source in the fluid equation

The increment $\Delta \mathbf{P}^s$ will differ based on growth law type. We calculate the increment for several different growth laws used in this work, based on a principle explained in Himpel et al. [17]. Briefly, the growth stretch ϑ is updated with a simple linear finite difference $\vartheta = \vartheta_n + \dot{\vartheta}\Delta t$. The incremental form of $\partial\vartheta/\partial\beta$ is obtained by taking the derivative of the update function with respect to the variables on which it depends, for example $\beta \in \{t, c\}$. For nonlinear growth, the resulting terms contain components of the local Newton-Raphson iterations.

Time-driven growth For time-dependent growth $\dot{\vartheta} = \alpha$, the finite difference yields an update function given by

$$\vartheta = \vartheta_n + \alpha\Delta t, \tag{S.54}$$

Note that the growth multiplier only varies with time, and thus, does not change within a corrector step, because time is fixed. During a predictor step, time changes, so the growth function also changes. Then for a predictor step, the increments in growth and the time derivative of growth are given by

$$\Delta\vartheta = \vartheta_{n+1} - \vartheta_n = \alpha\Delta t, \qquad \Delta\dot{\vartheta} = \dot{\vartheta}_{n+1} - \dot{\vartheta}_n = \alpha - \alpha = 0. \tag{S.55}$$

Then the increment in the growth source term is given by

$$\Delta(3\vartheta^2\dot{\vartheta}) = 6\vartheta(\Delta\vartheta)\dot{\vartheta} + 3\vartheta^2(\Delta\dot{\vartheta}) = 6\vartheta\dot{\vartheta}(\Delta\vartheta) + 0 = 6\vartheta\alpha^2\Delta t. \tag{S.56}$$

So during a predictor step, constant time-dependent growth is a body force; in the incremental finite element equation it is given by the forcing vector

$$\Delta\mathbf{P}_f^s = \int \mathbf{N}_f^T \left[6\vartheta\alpha^2\Delta t\right] dV_0. \tag{S.57}$$

Because this growth source is accounted for in the residual, this incremental term will only change the rate of convergence, and not the outcome of the Newton-Raphson iteration. Thus, the source terms may be neglected from the incremental equation.

Nonlinear concentration-driven growth For nonlinear concentration-dependent growth, (e.g., $\dot{\vartheta} = k_\vartheta(c - c_{\text{thresh}})$ for $k_\vartheta(\vartheta)$), the increment $\Delta\mathbf{P}^s$ becomes more complicated due to the nonlinearity of k_ϑ. Then, following the procedure from [17], take the derivative of the update $\vartheta = v_n + \dot{\vartheta}\Delta t$ with respect to c. Then

$$\frac{\partial\vartheta}{\partial c} = \left[\frac{\partial\dot{\vartheta}}{\partial c} + \frac{\partial\dot{\vartheta}}{\partial\vartheta}\frac{\partial\vartheta}{\partial c}\right]\Delta t. \tag{S.58}$$

Collecting like terms and rearranging,

$$\frac{\partial \vartheta}{\partial c} = \underbrace{\left(1 - \frac{\partial \dot{\vartheta}}{\partial \vartheta}\Delta t\right)}_{K_\vartheta}^{-1} \frac{\partial \dot{\vartheta}}{\partial c}\Delta t \tag{S.59}$$

where $K_\vartheta = [1 - (\partial k_\vartheta(\vartheta)/\partial\vartheta)\,(c - c_{\text{thresh}})\Delta t]$ is the local Newton-Raphson tangent for nonlinear time-dependent growth. We can directly calculate

$$\frac{\partial \dot{\vartheta}}{\partial c} = \frac{\partial k_\vartheta}{\partial c}(c - c_{\text{thresh}}) + k_\vartheta \frac{\partial(c - c_{\text{thresh}})}{\partial c} = 0 + k_\vartheta = k_\vartheta, \tag{S.60}$$

where $\partial k_\vartheta(\vartheta)/\partial c = 0$ because the scalar multiple k_ϑ does not directly depend on the concentration c.

Equation (S.59) simplifies to

$$\frac{\partial \vartheta}{\partial c} = \left(1 - \frac{\partial k_\vartheta(\vartheta)}{\partial \vartheta}(c - c_{\text{thresh}})\Delta t)\right)^{-1} k_\vartheta \Delta t = {K_\vartheta}^{-1} k_\vartheta \Delta t. \tag{S.61}$$

Finally, the increment in the source term is given by

$$\begin{aligned}\frac{\partial \overline{\mathcal{R}}_0}{\partial c}\Delta c = \frac{\partial[3\vartheta^2\dot{\vartheta}]}{\partial c}\Delta c &= 6\vartheta\frac{\partial \vartheta}{\partial c}\Delta c\dot{\vartheta} + 3\vartheta^2\frac{\partial \dot{\vartheta}}{\partial c}\Delta c \\ &= \left[6\vartheta {K_\vartheta}^{-1} {k_\vartheta}^2 (c - c_{\text{thresh}})\Delta t + 3\vartheta^2 k_\vartheta\right]\Delta c\end{aligned} \tag{S.62}$$

In this case as well, the increment in growth depends on the concentration, and so its contribution would be included in a $[\mathbf{k}^{G'}_{fcg}]$ matrix during the increment matrix; this is a secondary variable matrix. Evaluating the concentration at the Gauss point, the matrix becomes

$$[\mathbf{k}^{G'}_{fcg}]\Delta c^g = \left[\int \mathbf{N}_f^T \left[6\vartheta {K_\vartheta}^{-1} {k_\vartheta}^2 (c^g - c_{\text{thresh}})\Delta t + 3\vartheta^2 k_\vartheta\right] dV_0\right]\Delta c^g. \tag{S.63}$$

Stress-driven growth For stress-dependent growth ($\dot{\vartheta} = k_\vartheta(\text{tr}(\mathbf{M}^{\text{eff},e}) - M^{e,\text{equil}})$), the formulation is very similar to the solid-only material stress in [6, 17]; here we modify the tangent from the local Newton Raphson iterations for GMPHETS. Recall that during the global update, growth is fixed. Then

$$\left.\frac{\partial \vartheta}{\partial \mathbf{C}}\right|_{\mathbf{F}^g} = \frac{\partial \vartheta}{\partial \mathbf{C}^e} : \left.\frac{\partial \mathbf{C}^e}{\partial \mathbf{C}}\right|_{\mathbf{F}^g} = \frac{1}{\vartheta^2}\frac{k_\vartheta \Delta t}{K_\vartheta}\left(\frac{1}{2}\mathbf{C}^e : \mathbb{L}^e + \mathbf{S}^{\text{eff},e}\right), \tag{S.64}$$

from [6]. Also, we will need

$$\frac{\partial \dot{\vartheta}}{\partial \mathbf{C}} = k_\vartheta \frac{\partial[\text{tr}(\mathbf{M}^e) - M^{e,\text{equil}}]}{\partial \mathbf{C}^e} : \left.\frac{\partial \mathbf{C}^e}{\partial \mathbf{C}}\right|_{\mathbf{F}^g} = \frac{k_\vartheta}{\vartheta^2}\left(\frac{1}{2}\mathbf{C}^e : \mathbb{L}^e + \mathbf{S}^{\text{eff},e}\right), \tag{S.65}$$

as in [17]. Then the increment is given by

$$\frac{\partial \overline{\mathcal{R}}_0}{\partial \mathbf{C}}\Delta \mathbf{C} = \frac{\partial[3\vartheta^2\dot{\vartheta}]}{\partial \mathbf{C}}\Delta \mathbf{C} = 6\vartheta\frac{\partial \vartheta}{\partial \mathbf{C}}\Delta \mathbf{C}\dot{\vartheta} + 3\vartheta^2\frac{\partial \dot{\vartheta}}{\partial \mathbf{C}}\Delta \mathbf{C} = \left[6\vartheta\dot{\vartheta}\frac{\partial \vartheta}{\partial \mathbf{C}} + 3\vartheta^2\frac{\partial \dot{\vartheta}}{\partial \mathbf{C}}\right]\widehat{\mathbf{B}}_u \Delta \mathbf{u} \tag{S.66}$$

Here, growth depends on stress (which depends on strain and thus displacement). Thus, the contribution of the increment in growth would appear in the $[\mathbf{k}_{fu}]$ matrix during the increment, where there is now an extra growth contribution given by

$$[\mathbf{k}_{fu}^{\text{grow}}]\Delta\mathbf{u} = \left[\int \mathbf{N}_f^T \frac{\partial \overline{\mathcal{R}}_0}{\partial \mathbf{C}} \widehat{\mathbf{B}}_u dV_0\right] \Delta\mathbf{u}. \tag{S.67}$$

Incremental form of growth term in the species equation

This section derives the specific forms of the first part of the increment in the growth term from equation (S.45). This term changes based on the specific form of the growth law used. Note that the expression $\left(3\bar{\rho}^s\vartheta^2\dot{\vartheta}\right)$ is identical to that in the growth increment in the fluid conservation equation. Thus, we may reuse the derivations for the fluid to quickly arrive the increment in the species conservation equation.

Linear time-driven growth For linear time-dependent growth, the predictor increment has a forcing vector given by

$$\Delta\mathbf{P}_c^s = \int \mathbf{N}_c^T \bar{\rho}^s c^g \left[6\vartheta\alpha^2\Delta t\right] dV_0. \tag{S.68}$$

During a corrector step, time is fixed and this increment is zero.

Nonlinear concentration-driven growth For this case the growth increment depends on the concentration, and so its contribution would be included in a $[\mathbf{k}'_{ccg}]$ matrix; this is a secondary variable matrix given by

$$[\mathbf{k}'_{ccg}]\Delta c^g = \left[\int \mathbf{N}_c^T \bar{\rho}^s c^g \left[6\vartheta {K_\vartheta}^{-1} {k_\vartheta}^2 (c^g - c_{\text{thresh}})\Delta t + 3\vartheta^2 k_\vartheta\right] dV_0\right] \Delta c^g. \tag{S.69}$$

Nonlinear stress-driven growth In this case, the growth law depends on stress (which depends on strain and thus displacement). Thus, the contribution of the increment in growth would appear in the $[\mathbf{k}_{cu}]$ matrix during the increment, where there is now an extra growth contribution given by

$$[\mathbf{k}_{cu}^{\text{grow}}]\Delta\mathbf{u} = \left[\int \mathbf{N}_c^T \bar{\rho}^s c^g \frac{\partial \overline{\mathcal{R}}_0}{\partial \mathbf{C}} \widehat{\mathbf{B}}_u dV_0\right] \Delta\mathbf{u}, \tag{S.70}$$

and where $\dfrac{\partial \overline{\mathcal{R}}_0}{\partial \mathbf{C}}$ is given by equations (S.64) - (S.66).
