# Supplementary material for: A Finite Element Model for Mixed Porohyperelasticity with Transport, Swelling, and Growth
Source: PLoS One. 2016 Apr 14;11(4):e0152806. doi: 10.1371/journal.pone.0152806 (PMC4831841; doi:10.1371/journal.pone.0152806)
Supplement: S5 Appendix — (PDF) [file pone.0152806.s005.pdf]

S5 Appendix

Derivation of stresses and pore fluid pressure for time-dependent growth in an internally pressurized rigid cylinder

Consider an internally pressurized porohyperelastic cylinder made up of solid and fluid constituents. For simplicity, this problem will not include chemical transport. For a rigid cylinder, the conservation equations in the radially axisymmetric direction may be written as

$$\frac{1}{r}\frac{\partial[r\sigma_{rr}]}{\partial r} = 0, \tag{S.71}$$

and

$$\frac{1}{r}\frac{\partial[r j_r^{fr}]}{\partial r} - 3\vartheta^2\dot{\vartheta} = 0. \tag{S.72}$$

Note that the Eulerian form of these equations is used; σ_{ij} is the Cauchy stress and j_r^{fr} is the Eulerian relative fluid flux in the radial direction. The first closure equation is the stress-strain relationship that is modified by isotropic growth:

$$\sigma_{ij}^{\text{eff}} = J^{-1}F_{ik}S_{km}^{\text{eff}}F_{jm}, \qquad S_{km}^{\text{eff}} = \frac{1}{\vartheta^2}S_{km}^{\text{eff},e}, \qquad S_{km}^{\text{eff},e} = \frac{\partial W^{\text{eff}}}{\partial E_{km}^e} \tag{S.73}$$

where σ_{ij}^{eff} is the effective Cauchy stress, S_{km}^{eff} is the effective second Piola-Kirchhoff stress, $S_{km}^{\text{eff},e}$ is the effective, elastic second Piola-Kirchhoff stress, W^{eff} is the effective strain energy, and E_{km}^e is the elastic strain. The other closure equations are the effective stress principle in Eulerian coordinates

$$\sigma_{ij} = \sigma_{ij}^{\text{eff}} - p^f\delta_{ij}, \tag{S.74}$$

for the identity tensor δ_{ij}; and Darcy's law, given by

$$j_r^{fr} = -k_{rr}^{ff}\frac{\partial p^f}{\partial r} \tag{S.75}$$

where k_{rr}^{ff} is the radial diffusivity in the Eulerian frame. Note that for a porohyperelastic material, the Onsager equations reduce to Darcy's law.

Stresses Without growth ($\vartheta = 1$), the effective stresses in a rigid problem ($\mathbf{F} = \mathbf{I}$) are zero because the elastic strains are zero. With growth, on the other hand, zero deformation does not guarantee zero stresses. For a rigid problem, elastic deformation keeps the growing solid in the same configuration as the original.

For a rigid problem with isotropic growth, $\mathbf{F} = \mathbf{F}^e\mathbf{F}^g = \mathbf{I}$ results in

$$\mathbf{F}^e = \vartheta^{-1}\mathbf{I}, \qquad J^e = \det(\mathbf{F}^e) = \vartheta^{-3}, \qquad \mathbf{C}^e = \mathbf{F}^{eT}\mathbf{F}^e = \vartheta^{-2}\mathbf{I}. \tag{S.76}$$

The value of stress depends on the material law chosen for the effective stress. Using the values for a rigid problem from equation (S.76), the effective, elastic stress for isotropic growth of a Neo-Hookean type material is given by equation (71) as

$$\mathbf{S}^{\text{eff},e} = (\lambda\ln(J^e) - \mu)\mathbf{C}^{e-1} + \mu\mathbf{I} = (\lambda\ln(\vartheta^{-3}) - \mu)\vartheta^2\mathbf{I} + \mu\mathbf{I}. \tag{S.77}$$

Via the growth pullback from equation (59), the effective stress may be written as

$$\mathbf{S}^{\text{eff}} = \frac{1}{\vartheta^2}\mathbf{S}^{\text{eff},e} = \left(\lambda \ln(\vartheta^{-3}) - \mu + \frac{\mu}{\vartheta^2}\right)\mathbf{I}. \tag{S.78}$$

Recall that for a rigid problem, the second Piola-Kirchhoff stress is the same as the Cauchy stress because $\mathbf{S}^{\text{eff}} = J\mathbf{F}^{-1}\boldsymbol{\sigma}^{\text{eff}}\mathbf{F}^{-T}$ and rigidity implies $\mathbf{F} = \mathbf{I}$ and $J = 1$.

Pore fluid pressure for time-dependent growth on a rigid axisymmetric cylinder Substituting Darcy's Law (S.75) into the fluid conservation equation (S.72) yields

$$\frac{1}{r}\frac{\partial}{\partial r}\left[-rk_{rr}^{ff}\frac{\partial p^f}{\partial r}\right] - 3\vartheta^2\dot{\vartheta} = 0 \tag{S.79}$$

For simplicity, assume that the permeability is constant in space. For time-dependent growth (i.e., $\dot{\vartheta} = \alpha$ whence $\vartheta = \alpha t + 1$), the growth term is also constant in space. Then rearranging terms and integrating twice in r,

$$p^f = -\frac{3\vartheta^2\dot{\vartheta}}{4k^{ff}}r^2 + \frac{\tilde{A}}{k^{ff}}\log(r) + B, \tag{S.80}$$

for constants of integration $\tilde{A}, B$. To simplify notation, define constant $A = \tilde{A}/k^{ff}$ and growth-dependent scalar

$$\tilde{D} = -\frac{3\vartheta^2\dot{\vartheta}}{4k^{ff}}. \tag{S.81}$$

Then the pore fluid pressure may be written simply as

$$p^f(r) = \tilde{D}r^2 + A\log(r) + B. \tag{S.82}$$

The boundary conditions determine coefficients A, B. For an internally pressurized cylinder $r_1 \leq r \leq r_2$ such that $p^f(r_1) = P$ and $p^f(r_2) = 0$, the pore fluid pressure becomes

$$p^f(r) = \tilde{D}(r^2 - r_2^2) + \left(P - \tilde{D}(r_1^2 - r_2^2)\right)\frac{\log(r/r_2)}{\log(r_1/r_2)}. \tag{S.83}$$

Substituting in the definition for $\tilde{D}$,

$$p^f(t, r, \vartheta) = -\frac{3\vartheta^2\dot{\vartheta}}{4k^{ff}}(r^2 - r_2^2) + \left(P + \frac{3\vartheta^2\dot{\vartheta}}{4k^{ff}}(r_1^2 - r_2^2)\right)\frac{\log(r/r_2)}{\log(r_1/r_2)}. \tag{S.84}$$

In the case of no growth ($\dot{\vartheta} = 0; \vartheta = 0$), equation (S.84) simplifies to

$$p^f(t, r, \vartheta) = P\frac{\log(r/r_2)}{\log(r_1/r_2)}, \tag{S.85}$$

which matches the solution calculated for an internally-pressurized linear poroelastic cylinder, with no growth (not shown).
