# Supplementary material for: A Finite Element Model for Mixed Porohyperelasticity with Transport, Swelling, and Growth
Source: PLoS One. 2016 Apr 14;11(4):e0152806. doi: 10.1371/journal.pone.0152806 (PMC4831841; doi:10.1371/journal.pone.0152806)
Supplement: S6 Appendix — (PDF) [file pone.0152806.s006.pdf]

S6 Appendix

Table 1. Densities used in GMPHETS theory

$\rho_{0,\text{init}}$	initial total density in reference configuration	initial total mass of the material (initial solid mass plus initial fluid mass) divided by original volume dV_0
ρ_0	grown total density in reference configuration	total grown mass of the material (grown solid mass plus grown fluid mass) divided by original volume dV_0
$\widehat{\rho}_0$	grown total density in intermediate growth configuration	total grown mass of the material (grown solid mass plus grown fluid mass) divided by intermediate growth volume $d\widehat{V}$
ρ	grown total density in final configuration	total grown mass of the material (grown solid mass plus grown fluid mass) divided by final volume dV
ρ_T^s	true density of solid	mass of solid divided by volume of solid; for an incompressible solid this quantity will be the same in any real, observable configuration
ρ_T^f	true density of fluid	mass of fluid divided by volume of fluid; for an incompressible fluid this quantity will be the same in any real, observable configuration
$\rho_{0,\text{init}}^s$	initial apparent solid density in reference configuration	initial solid mass divided by original volume dV_0
$\rho_{0,\text{init}}^f$	initial apparent fluid density in reference configuration	initial fluid mass divided by original volume dV_0
ρ_0^s	grown apparent solid density in reference configuration	grown mass of the solid divided by original volume dV_0
ρ_0^f	grown apparent fluid density in reference configuration	grown mass of the fluid divided by original volume dV_0
$\widehat{\rho}_0^s$	grown apparent solid density in intermediate growth configuration	grown mass of the solid divided by intermediate growth volume $d\widehat{V}$
$\widehat{\rho}_0^f$	grown apparent fluid density in intermediate growth configuration	grown mass of the solid divided by intermediate growth volume $d\widehat{V}$
ρ^s	grown apparent solid density in final configuration	grown mass of the solid divided by final volume dV
ρ^f	grown apparent fluid density in final configuration	grown mass of the fluid divided by final volume dV
$\rho_0^{s^*}$	solid density preserved during growth	either true density of solid (solid-only growth); or initial apparent solid density in reference configuration (solid/fluid growth)
$\rho_0^{f^*}$	fluid density preserved during growth	initial apparent fluid density in reference configuration (solid/fluid growth)
$\bar{\rho}^s$	normalized growth density of solid in original configuration	solid density preserved during growth divided by true density of solid
$\bar{\rho}^f$	normalized growth density of fluid in original configuration	fluid density preserved during growth divided by true density of fluid
